# CD38‐Specific CAR Integrated into *CD38* Locus Driven by Different Promoters Causes Distinct Antitumor Activities of T and NK Cells

**DOI:** 10.1002/advs.202207394

**Published:** 2023-07-23

**Authors:** Chan Liao, Yajie Wang, Yanjie Huang, Yanting Duan, Yan Liang, Jiangqing Chen, Jie Jiang, Kai Shang, Chun Zhou, Ying Gu, Nan Liu, Xun Zeng, Xiaofei Gao, Yongmin Tang, Jie Sun

**Affiliations:** ^1^ Department of Hematology‐oncology Children's Hospital Zhejiang University School of Medicine Pediatric Leukemia Diagnostic and Therapeutic Technology Research Center of Zhejiang Province National Clinical Research Center for Child Health Hangzhou 310003 China; ^2^ Liangzhu Laboratory Zhejiang University Medical Center Hangzhou 311121 China; ^3^ Bone Marrow Transplantation Center of the First Affiliated Hospital and Department of Cell Biology Zhejiang University School of Medicine Hangzhou 310058 China; ^4^ Institute of Hematology Zhejiang University & Zhejiang Engineering Laboratory for Stem Cell and Immunotherapy Hangzhou 310058 China; ^5^ Key Laboratory of Structural Biology of Zhejiang Province School of Life Sciences Westlake University Hangzhou 310058 China; ^6^ School of Basic Medical Sciences Fudan University Shanghai 200032 China; ^7^ School of Public Health and Sir Run Run Shaw Hospital Zhejiang University School of Medicine Hangzhou 310058 China; ^8^ Institute of Genetics, Zhejiang University and Department of Genetics Zhejiang University school of medicine Hangzhou 310058 China; ^9^ State Key Laboratory for Diagnosis and Treatment of Infectious Diseases First Affiliated Hospital Zhejiang University School of Medicine Hangzhou 310058 China

**Keywords:** adoptive cell therapy, CAR, CD38, CRISPR/Cas9, gene editing, natural killer cells, T‐cell acute lymphoblastic leukemia

## Abstract

The robust and stable expression of CD38 in T‐cell acute lymphoblastic leukemia (T‐ALL) blasts makes CD38 chimeric antigen receptor (CAR)‐T/natural killer (NK) a potential therapy for T‐ALL. However, CD38 expression in normal T/NK cells causes fratricide of CD38 CAR‐T/NK cells. Here a “2‐in‐1” gene editing strategy is developed to generate fratricide‐resistant locus‐specific CAR‐T/NK cells. CD38‐specific CAR is integrated into the disrupted *CD38* locus by CRISPR/Cas9, and CAR is placed under the control of either endogenous CD38 promoter (*CD38* ^KO/KI^) or exogenous EF1α promoter (*CD38* ^KO/KI^EF1α). CD38 knockout reduces fratricide and allows the expansion of CAR‐T cells. Meanwhile, *CD38* ^KO/KI^EF1α results in higher CAR expression than *CD38* ^KO/KI^ in both CAR‐T and CAR‐NK cells. In a mouse T‐ALL model, *CD38* ^KO/KI^EF1α CAR‐T cells eradicate tumors better than *CD38* ^KO/KI^ CAR‐T cells. Surprisingly, *CD38* ^KO/KI^ CAR‐NK cells show superior tumor control than *CD38* ^KO/KI^EF1α CAR‐NK cells. Further investigation reveals that endogenous regulatory elements in NK cells lead to higher expression of CD38 CAR than in T cells, and the expression levels of CAR affect the therapeutic outcome of CAR‐T and CAR‐NK cells differently. Therefore, these results support the efficacy of CD38 CAR‐T/NK against T‐ALL and demonstrate that the “2‐in‐1” strategy can resolve fratricide and enhance tumor eradication, paving the way for clinical translation.

## Graphical abstract

A new “2‐in‐1” strategy is developed to insert CD38 chimeric antigen receptor (CAR) at the *CD38* locus by CRISPR/Cas9, which can resolve fratricide and enhance tumor eradication. With different promoters at the *CD38* locus, the authors unexpectedly discover that the EF1α promoter in CAR‐T is better, while *CD38* endogenous promoter in CAR‐natural killer (NK) is superior.

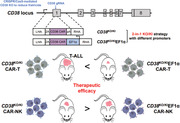

## 1 Introduction

T‐cell acute lymphoblastic leukemia (T‐ALL) is an aggressive hematological cancer caused by the stepwise transformation of T lymphoblasts during T‐cell development in the thymus. It is phenotypically and genetically heterogeneous, usually related to genetic alterations or mutations of transcription factors associated with T cell development.^[^ ^1^, ^2^, ^3^ ^]^ T‐ALL accounts for 10–15% of pediatric and 20–25% of adult ALL cases.^[^ ^4^ ^]^ Although the survival rate of most patients has improved significantly after receiving intensive chemotherapy, the treatment of relapsed/refractory (r/r) T‐ALL still faces serious challenges. Furthermore, the early T‐cell precursor subtype of T‐ALL (ETP‐ALL), a high‐risk subset of T‐ALL, which accounts for 10–15% of T‐ALL, has been shown to have a poorer clinical outcome in adult patients.^[^ ^5^ ^]^ Unfortunately, few therapies have been approved for T‐ALL since the approval of nelarabine by the US Food and Drug Administration (FDA) in 2005.^[^ ^6^ ^]^ So it is urgent to explore new therapies.

As a promising immunotherapy, chimeric antigen receptor‐T (CAR‐T)‐cell therapy targeting B‐cell malignancies has made remarkable achievements in the clinic. Many patients with refractory or relapsed B‐cell malignancies have achieved complete remission after receiving T cells that are redirected with CARs targeting the B‐cell antigen CD19.^[^ ^7^, ^8^, ^9^ ^]^ However, extending the success of CAR T cells to treat T‐cell malignancies, such as T‐ALL, has been challenging due to the shared expression of targetable antigens between normal and malignant T cells, which will result in fratricide and limit the expansion of CAR‐T cells.^[^ ^10^ ^]^ In addition, targeting an antigen regularly expressed on normal T cells will result in T cell aplasia, leading to profound immunosuppression.^[^ ^11^ ^]^ Furthermore, it is hard to isolate healthy T cells from malignant T cells based on surface markers for the production of CAR‐T cells, which can cause product contamination and subsequent CAR‐modification of tumor cells.^[^ ^12^, ^13^ ^]^ All of the above problems greatly limit the application of CAR‐T cell therapy in T‐ALL.

So far, many studies have explored CAR‐T therapies that target CD7, CD5, CD4, CD1a, and T cell receptors β constant 1 (TRBC1) in the treatment of T‐ALL.^[^ ^14^, ^15^, ^16^, ^17^, ^18^ ^]^ The expression of CD5 on the surface of malignant T cells but not hematopoietic stem cells makes it a good target for T‐ALL. However, the expression level of CD5 is often low on the surface of ETP‐ALL,^[^ ^5^ ^]^ necessitating the search for new targets. Highly expressed CD7 in T‐ALL blasts, including ETP‐ALL, is considered a better target for ETP‐ALL,^[^ ^5^ ^]^ and many preclinical studies^[^ ^19^, ^20^, ^21^ ^]^ and clinical trials^[^ ^10^ ^]^ of CD7 CAR‐T cells for T‐ALL are ongoing. In addition, when patients experienced CD19‐negative relapse after CD19 CAR‐T therapy, CAR‐T cells targeting CD22 or BAFF‐R can eliminate the relapsed tumor that was resistant to CD19 CAR‐T cells.^[^ ^22^, ^23^ ^]^ Therefore, finding new CAR targets for T‐ALL would be useful in the clinic.

CD38 is a single‐pass type II transmembrane glycoprotein that is involved in cell adhesion, signal transduction, calcium regulation, and many other functions. CD38 has been found to express in many hematological malignancies, including multiple myeloma (MM), ALL, chronic lymphocytic leukemia (CLL), and acute myeloid leukemia (AML), making it an attractive target for immunotherapy.^[^ ^24^ ^]^ In addition, CD38 is regularly and stably expressed at high levels in tumor cells of adult and pediatric patients with T‐ALL.^[^ ^25^ ^]^ Daratumumab, a monoclonal antibody (mAb) targeting CD38, which was approved by the FDA in 2015 to treat MM, has shown encouraging results for the treatment of T‐ALL in preclinical studies. Currently, two clinical trials of Daratumumab for the treatment of patients with r/r T‐ALL are being conducted.^[^ ^26^ ^]^ The safety and promising efficacy of anti‐CD38 mAb‐based treatments have prompted the idea to use CD38‐specific CAR‐T or CAR‐NK therapies to treat T‐ALL. Since CD38 is also expressed by T and NK cells leading to fratricide, a previous study used an anti‐CD38 antibody to reduce fratricide through cross‐linking anti‐CD38 CAR with intrinsic CD38.^[^ ^27^ ^]^ Alternatively, gene editing by CRISPR/Cas9 was used to disrupt the *CD38* gene of NK cells to reduce fratricide during the treatment of AML.^[^ ^28^ ^]^ Besides T and NK cells, CD38 is also expressed in other hematological cells like precursor B cells, plasma cells, and non‐hematological tissue. It has been reported that affinity optimization of CD38 CAR could reduce “on‐target, off‐tumor” effects when targeting MM.^[^ ^29^ ^]^ Despite the success of CD38 CAR targeting MM, AML, and B‐ALL, the application of CD38 CAR‐T cells in the treatment of T‐ALL is scarce.

CAR‐T cell therapy targeting T cell malignancies requires the separation of healthy T cells from malignant T cells. Since there were no markers to easily differentiate healthy and malignant T cells, malignant T cells can be modified with CAR and contaminate CAR‐T products. Some approaches have been used to overcome the challenge. One method is to use allogeneic T cells from healthy donors as the cell source for generating CAR‐T cells. To reduce graft versus host diseases (GvHD), expression of the endogenous αβ T cell receptor （αβTCR） in allogeneic CAR T cells must be blocked unless the donor has matched human leukocyte antigens (HLAs). Using CRISPR/Cas9 to disrupt both *CD7* and T‐cell receptor α constant (*TRAC*) locus, one study generated “fratricide‐resistant, allo‐tolerant” CAR‐T cells targeting CD7, which could eliminate T‐ALL cells without developing GvHD.^[^ ^19^ ^]^ In addition to allogeneic T cells, NK cells can also be used as an alternative cell source to solve the problem. Several groups have published studies targeting T cell malignancies with CAR NK‐92 cells, an Interleukin (IL2)‐dependent NK‐lymphoma‐derived cell line.^[^ ^30^, ^31^, ^32^ ^]^ For target antigens that are also expressed in primary NK cells, one study applied CRISPR/Cas9 gene editing to disrupt the *CD38* gene of NK cells to reduce fratricide and then transduced CD38 CAR into NK cells and tested their efficacy against AML.^[^ ^28^ ^]^

In this study, we investigated the feasibility of targeting T‐ALL using CD38 CAR‐ T/NK cells. We utilized the CRISPR/Cas9 gene editing platform to disrupt the expression of CD38 prior to CAR transduction, which prevented fratricide and promoted the expansion of CD38 CAR‐T cells. We then designed an efficient “2‐in‐1” KO/KI (knockout and knockin) strategy to integrate the CD38 CAR into the *CD38* locus while knocking out the *CD38* gene. To determine the importance of the expression level of the CD38 CAR for CD38 CAR‐T/NK cell functions, we tested two designs within the 2‐in‐1 framework, one using the endogenous *CD38* locus regulatory elements and the other using an exogenous EF1α promoter. We found that T cells with CD38 CAR inserted into the *CD38* locus and driven by EF1α promoter eliminated tumors more efficiently in vitro and in a mouse model of T‐ALL. On the contrary, NK cells expressing CD38 CAR under the control of *CD38* endogenous promoters showed better tumor control. Further investigation revealed that NK cells expressed higher levels of CD38 than T cells, and CD38 CAR expression was also higher in NK cells. Thus, it appears that the regulation of *CD38* locus in T and NK cells was different, resulting in different promoter strength requirements for optimal CAR‐T/NK efficacy. To summarize, we demonstrated the feasibility of CD38 CAR‐T/NK cells targeting T‐ALL. Our unique “2‐in‐1” strategy could resolve fratricide and enhance tumor rejection, which supports future clinical studies. Moreover, we unexpectedly found that CD38 CAR integrated into the *CD38* locus regulated by endogenous and exogenous promoters caused the distinct antitumor activity of T and NK cells, suggesting the expression level of CAR in different cells could be fine‐tuned to maximize its therapeutic potential.

## 2 Results

### 2.1 CD38 Expression on T Cells Led to Fratricide and Limited Expansion of CD38 CAR‐T Cells

**Figure 1:**
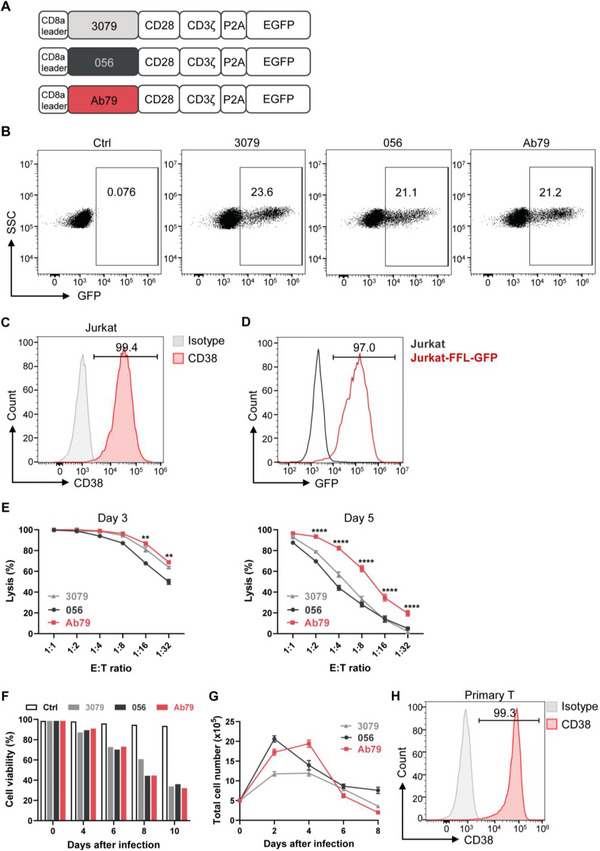
CD38 CAR expression on T cells induced fratricide and limited expansion of CD38 CAR‐T cells. A) Schematic of three CD38 CAR constructs. B) The expression level of three CARs measured by flow cytometry on day 3 post‐transduction. Untransduced T cells were used as the control. Ctrl, Control. Representative data from one of the three independent experiments are shown. C) Expression of CD38 on Jurkat cells was detected by flow cytometry. Isotype was used as the control. D) Jurkat‐expressing firefly luciferase (FFLuc)‐enhanced green fluorescent protein (EGFP) constructed by infecting Jurkat cells with a lentiviral vector containing FFLuc‐EGFP. E) Cytotoxicity of three kinds of CAR‐T cells 3 days after transduction (left) and 5 days after transduction (right) against FFLuc‐expressing Jurkat cells at various effector‐to‐target (E:T) ratios using an 18 h bioluminescence assay. Data represent the mean ± SEM of triplicates from one of the three independent experiments. Statistical analysis was performed between Ab79 and 3079 groups. F) Cell viability of T cells after CAR transduction in vitro for 10 days. G) Expansion of T cells after transduction with CD38 CAR in vitro for 8 days. Data represent the mean ± SEM of triplicates. H) Expression of CD38 on primary T cells was detected by flow cytometry. Isotype was used as the control. *p < 0.05, **p < 0.01, ***p < 0.001, and ****p < 0.0001; ns, not significant.

We generated three CAR constructs with CD38‐specific single‐chain variable fragments (scFv) derived from 056, Ab79, and 3079 clones of CD38 antibodies,^[^ ^33^ ^]^ which had variable affinities (Table S1, Supporting Information). The CAR constructs contained the CD28 co‐stimulation domain and the CD3ζ signaling domain (**Figure** **1**A). All CAR‐T cells were produced with similar transduction efficiencies (Figure 1B). We chose the CD38‐positive T‐ALL Jurkat cell line as target cells and constructed Jurkat‐luciferase cells (Figure 1C,D). Ab79 CAR‐T cells showed the highest cytotoxicity (Figure 1E). However, the cell viability of three CAR‐T cells decreased from more than 95% to about 30%, 10 days after CAR transduction (Figure 1F). Also, the cells did not expand well, with the cell number increasing only in the first few days and then decreasing dramatically later (Figure 1G). This was likely caused by fratricide between CD38 CAR‐T cells, which is related to the expression of CD38 on the surface of T cells (Figure 1H). Based on the above data, we selected Ab79 scFv, which displayed better cytotoxic function, for the construction of CD38 CAR in the following experiments.

### 2.2 CD38‐Disrupted *TRAC* Locus‐Targeted CD38 CAR‐T Cells Exhibited Better In Vitro Functions than Retrovirally Transduced CAR‐T Cells

**Figure 2:**
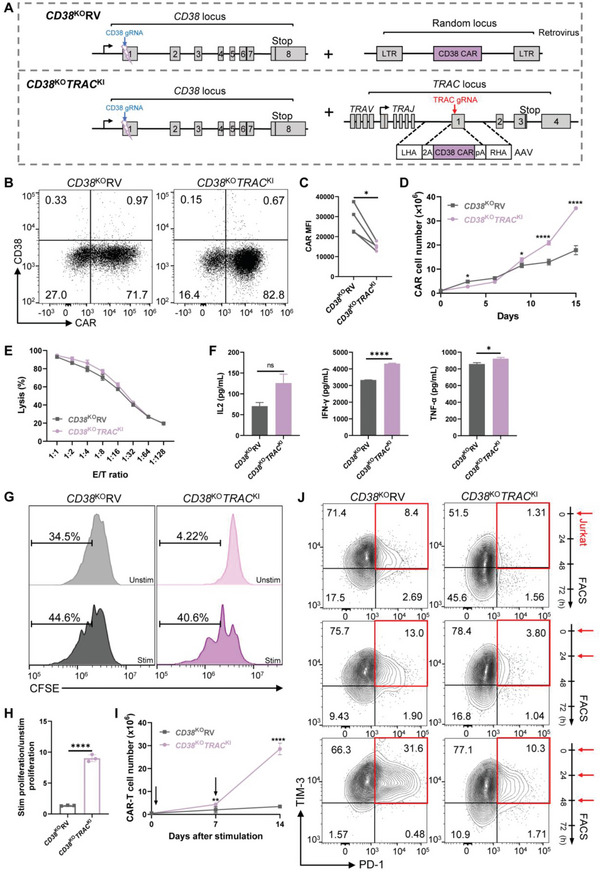
CD38‐disrupted *TRAC*‐encoded CD38 CAR‐T cells displayed better in vitro functions compared to retrovirally transduced CAR‐T cells. A) Schematic of CRISPR/Cas9‐targeted CD38 gene KO and CD38 CAR gene integration. Top, *CD38* ^KO^RV, disrupted *CD38* locus by CRISPR/Cas9 and random integration of CAR gene by retrovirus; Bottom, *CD38* ^KO^ *TRAC* ^KI^, disrupted *CD38* locus by CRISPR/Cas9 and site‐specific integration of CAR gene by rAAV6 containing the P2A‐CAR‐polyA cassette flanked by homology arms. gRNA, guide RNA; 2A, the self‐cleaving Porcine teschovirus 2A sequence; pA, bovine growth hormone polyA sequence; LHA and RHA, left and right homology arm. B) Representative dot plots showing expression of CD38 CAR in T cells 4 days after generation with the optimized protocol. Numbers indicate the percentage of cells in each quadrant. Representative results from at least three donors. C) CAR mean fluorescence intensity (MFI) 4 days after gene transfer (*n* = 4 donors). The paired comparison within each donor was connected by a line. D) Expansion of T cells after transduction with CD38 CAR in vitro. Cells were cultured in normal conditions after CAR transduction and counted every 3 days. Data represent the mean ± SEM of triplicates. E) The cytotoxic activity of both CAR T cells upon co‐culture with target cells. Data represent the mean ± SEM of triplicates from one of the three independent experiments. F) Concentrations of cytokine secreted by both CAR‐T cells. Data represent the mean ± SEM of triplicates from one of the three independent experiments. G) Proliferation of CAR‐T cells analyzed by carboxyfluorescein succinimidyl ester (CFSE) assay. Unstim represents CAR‐T cells without stimulation by Jurkat cells and stim represents CAR‐T cells stimulated by Jurkat cells for 72 h. H) CAR‐T cell antigen‐dependent proliferation fold calculated by stim/unstim ratio. Data represent the mean ± SEM of triplicates. I) Cumulative cell numbers of CAR T cells upon weekly stimulation with target cells. Arrows indicate stimulation time points. Data represent the mean ± SEM of triplicates. J) carboxyfluorescein succinimidyl ester FACS analysis of exhaustion makers (PD‐1 and TIM‐3) on CAR‐T cells stimulated 1, 2, or 3 times by target cells. *p < 0.05, **p < 0.01, ***p < 0.001, and ****p < 0.0001; ns, not significant.

To knockout the *CD38* gene with CRISPR/Cas9, we used the guide RNA (gRNA) targeting the first exon of the *CD38* gene from Naeimi Kararoudi et al.^[^ ^34^ ^]^ and verified that the knockout efficiency was above 78% in different donors (Figure S1A,B, Supporting Information). As Eyquem et al.^[^ ^35^ ^]^ found that targeting CD19‐specific CAR to the *TRAC* locus enhanced T cell potency, we investigated whether inserting CD38 CAR to the *TRAC* locus would also be beneficial in the context of *CD38* disruption. Our results showed that both *CD38* and *TRAC* genes could be efficiently disrupted, and the double KO efficiency exceeded 60% (Figure S1C, Supporting Information). Next, CARs were delivered to the *TRAC* locus of edited T cells using a recombinant adeno‐associated virus (rAAV) vector encoding CD38 CAR to generate *CD38* ^KO^ *TRAC* ^KI^ CAR‐T cells (**Figure** **2**A). Meanwhile, we transduced T cells with CD38 CAR‐encoding retrovirus after *CD38* disruption to generate *CD38* ^KO^RV CAR‐T cells (Figure 2A and Figure S1D, Supporting Information). Both CAR‐T cells had similar CAR^+^ percentages (Figure 2B), but *CD38* ^KO^RV CAR‐T cells displayed nearly twofold higher mean fluorescent intensity (MFI) of CAR than *CD38* ^KO^ *TRAC* ^KI^ cells (Figure 2C). And *CD38* ^KO^RV CAR‐T cells had around 10.5% cells with phosphorylated CD3ζ in the absence of antigen stimulation, while *CD38* ^KO^ *TRAC* ^KI^ CAR‐T had less than 0.5% (Figure S2A, Supporting Information). With the disruption of *CD38*, we observed continuous expansion of *CD38* ^KO^RV CAR‐T cells (Figure 2D), different from the limited expansion of CAR‐T cells with CD38 expression (Figure 1G). *CD38* ^KO^ *TRAC* ^KI^ CAR‐T cells also showed continuous expansion and expanded even better than *CD38* ^KO^RV CAR‐T cells (Figure 2D; and Figure S2B, Supporting Information). These results indicated that CD38 knockout in T cells could prevent fratricide, thereby promoting the expansion of CAR‐T cells.

We next evaluated their cytotoxic effect and observed equipotent tumor lysis at all tested E:T ratios (Figure 2E and Figure S2C, Supporting Information). Cytokine release assay revealed that *CD38* ^KO^ *TRAC* ^KI^ CAR‐T cells produced more tumor necrosis factor‐α (TNF‐α) and interferon‐γ (IFN‐γ) than *CD38* ^KO^RV CAR‐T cells after co‐culturing with Jurkat cells (Figure 2F and Figure S2D, Supporting Information). Using CFSE assay, we found that in the absence of target cells, *CD38* ^KO^RV CAR‐T cells proliferated more significantly compared to *CD38* ^KO^ *TRAC* ^KI^ CAR‐T cells (Figure 2G; and Figure S2E,F Supporting Information). However, *CD38* ^KO^ *TRAC* ^KI^ CAR‐T cells showed a much higher antigen‐induced proliferation capacity calculated by the stim proliferation/unstim proliferation ratio (Figure 2H; and Figure S2G, Supporting Information). In addition, *CD38* ^KO^ *TRAC* ^KI^ CAR‐T cells exceeded *CD38* ^KO^RV CAR‐T cells in the total CAR‐T cell number after consecutive antigen stimulations (Figure 2I; and Figure S2H, Supporting Information). We also examined the expression of exhaustion makers, PD‐1 and TIM‐3, on CD38 CAR‐T cells after they were stimulated with Jurkat cells as indicated. With increasing times of stimulation, the proportion of PD‐1^+^TIM‐3^+^ CAR‐T cells in both CAR‐T cells increased. No matter how many times the cells were stimulated, *CD38* ^KO^RV CAR‐T cells always had a higher percentage of PD‐1^+^TIM‐3^+^ cells than *CD38* ^KO^ *TRAC* ^KI^ CAR‐T cells. And when stimulated three times, the TIM‐3 expression level of *CD38* ^KO^RV CAR‐T cells was 30% higher than *CD38* ^KO^ *TRAC* ^KI^ CAR‐T cells (Figure 2J; and Figure S2I, Supporting Information). Together these results indicated that CD38 CAR‐T cells generated by targeting CAR into the *TRAC* locus demonstrated superior function in vitro than CD38 CAR‐T cells generated from retroviral transduction.

### 2.3 “2‐in‐1” KO/KI Strategies were Developed to Generate CD38 CAR‐T Cells

**Figure 3:**
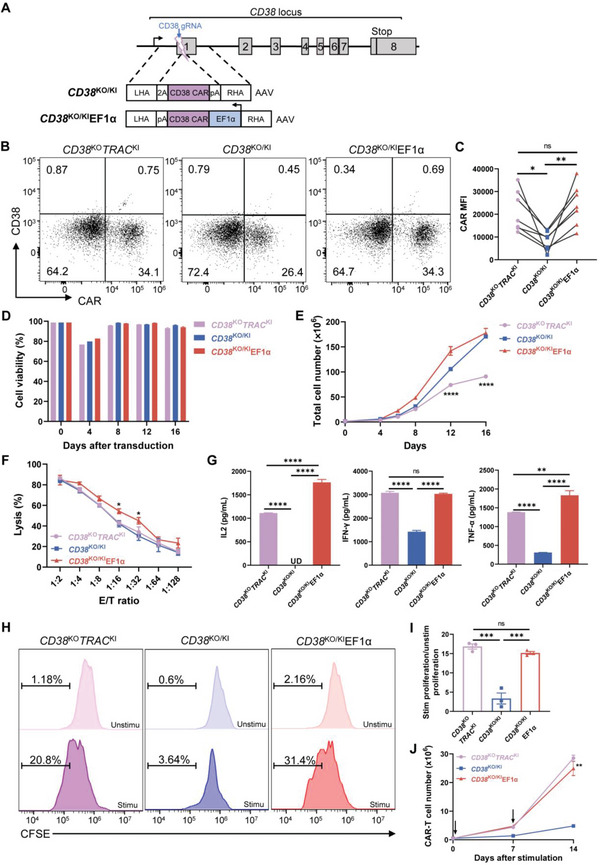
In vitro functions of CD38 CAR‐T cells generated by “2‐in‐1” KO/KI strategies. A) Schematic of CRISPR/Cas9‐targeted CD38 CAR gene integration into the *CD38* locus. Top, *CD38* locus; Middle, rAAV6 containing the P2A‐CAR‐polyA cassette flanked by homology arms; Bottom, rAAV6 containing the CD38 CAR coding sequence in the reverse orientation, under the control of an exogenous promoter, EF1α. B) Representative dot plots showing the expression of CD38 and CD38 CAR in T cells 4 days after gene editing. Representative results from at least three donors. C) CAR MFI 4 days after gene transduction (*n* = 7 donors). The paired comparison within each donor was connected by a line. D) Cell viability of T cells after CAR transduction in vitro for 16 days. Data represent the mean ± SEM of triplicates. E) Expansion of CAR‐T cells after CAR transduction in vitro for 16 days. Data represent the mean ± SEM of triplicates. Statistical analysis was performed between *CD38* ^KO^ *TRAC* ^KI^ and *CD38* ^KO/KI^ groups. F) Cytotoxic activity of CAR‐T cells against target cells using an 18 h bioluminescence assay. Data represent the mean ± SEM of triplicates from one of the three independent experiments. Statistical analysis was performed between *CD38* ^KO^ *TRAC* ^KI^ and *CD38* ^KO/KI^EF1α groups. G) Cytokine secretion by CAR‐T cells was detected after stimulation by target cells. UD, Undetected. Data represent the mean ± SEM of triplicates from one of the three independent experiments. H) Proliferation of CAR‐T cells analyzed by CFSE assay. Unstim represents CAR‐T cells without stimulation by target cells and stim represents CAR‐T cells stimulated by Jurkat cells for 72 h. I) CAR‐T cell antigen‐dependent proliferation fold calculated by stim/unstim ratio. Data represent the mean ± SEM of triplicates. J) Cumulative cell numbers of CAR T cells upon weekly stimulation with target cells. Arrows indicate stimulation time points. Data represent the mean ± SEM of triplicates. Statistical analysis was performed between *CD38* ^KO^ *TRAC* ^KI^ and *CD38* ^KO/KI^EF1α. *p < 0.05, **p < 0.01, ***p < 0.001, and ****p < 0.0001; ns, not significant.

Generating *CD38* ^KO^ *TRAC* ^KI^ CAR‐T cells required two types of gRNAs, which increased “off‐target” risk and the cost of experiments. In addition, more RNPs were required for simultaneous disruption of the *TRAC* and *CD38* (*CD38* ^KO^ *TRAC* ^KO)^ in T cells to achieve high double knockout efficiency. Our results showed that *CD38* ^KO^ *TRAC* ^KO^ T cells did not expand as well as *CD38* ^KO^ T cells (Figure S3A, Supporting Information). So, we devised a “2‐in‐1” KO/KI strategy to insert the CD38 CAR at the *CD38* locus and disrupt the *CD38* gene at the same time. What is more, to control the expression of CAR, we generated two constructs with different promoters. One design was to use a 2A self‐cleavage peptide preceding inserted CD38 CAR to put the CAR under the control of the endogenous *CD38* promoter just as in the *TRAC* locus, namely *CD38* ^KO/KI^ CAR‐T. The other used an exogenous EF1α promoter to control CD38 CAR expression, namely *CD38* ^KO/KI^EF1α CAR‐T (**Figure** **3**A). So we generated three T cells expressing CD38 CAR at defined genomic loci and under the control of different promoters for subsequent functional comparison: *CD38* ^KO^ *TRAC* ^KI^, *CD38* ^KO/KI^, and *CD38* ^KO/KI^EF1α CAR‐T cells.

The knockin efficiencies of three CARs were comparable, all around 30% (Figure 3B). But, as designed, the three CARs showed different MFIs, with the MFI of *CD38* ^KO^ *TRAC* ^KI^ CAR and *CD38* ^KO/KI^EF1α CAR similar, twofold higher than that of *CD38* ^KO/KI^ CAR (Figure 3C). Even though *CD38* knockout efficiency could not reach 100% (Figure S1A,B, Supporting Information), there were no CD38‐positive T cells 4 days after CAR introduction, suggesting that residual CD38^+^ T cells should have been eliminated by CD38 CAR‐T cells, further confirmed the functionality of these CAR‐T cells (Figure 3B). Even though there was a transient decrease in cell viability 4 days after CAR introduction, the cell viability recovered and remained stable afterward (Figure 3D). Due to the short‐lived fratricide, there was a slow increase in cell numbers for the first four days, after which the cells expanded faster (Figure 3E).

Next, cytotoxicity assay showed that three types of CAR‐T cells effectively killed Jurkat cells (Figure 3F; and Figure S3B, Supporting Information). However, *CD38* ^KO/KI^ CAR‐T cells produced significantly lower levels of cytokines than *CD38* ^KO^ *TRAC* ^KI^ and *CD38* ^KO/KI^EF1α CAR‐T cells when stimulated with Jurkat cells (Figure 3G). Data from two additional donors confirmed this finding (Figure S3C, Supporting Information). Even though the baseline proliferation of these three CD38 CAR‐T cells was similarly low in the absence of target cells, *CD38* ^KO/KI^ CAR‐T cells showed the lowest stim proliferation/unstim proliferation ratio compared to *CD38* ^KO^ *TRAC* ^KI^ and *CD38* ^KO/KI^EF1α cells after antigen stimulation (Figure 3H,I). In addition, *CD38* ^KO/KI^ CAR‐T cells showed the slowest growth in cell number after two consecutive antigen stimulations (Figure 3J). The proportion of PD‐1^+^LAG‐3^+^ CAR‐T cells in all three CAR‐T cells increased with repeated stimulation. And *CD38* ^KO/KI^ CAR‐T cells showed a lower proportion of PD‐1^+^LAG‐3^+^ CAR‐T cells when stimulated three times (Figure S3D, Supporting Information). Therefore, these CD38 CAR‐T cells demonstrated potent cytotoxicity against tumor cells in vitro, while *CD38* ^KO/KI^EF1α CAR‐T cells performed better in terms of cytokine secretion and proliferation than *CD38* ^KO/KI^ CAR‐T cells.

### 2.4 CD38 CAR‐T Cells Recognize and Eliminate Primary T‐ALL Cells In Vitro

**Figure 4:**
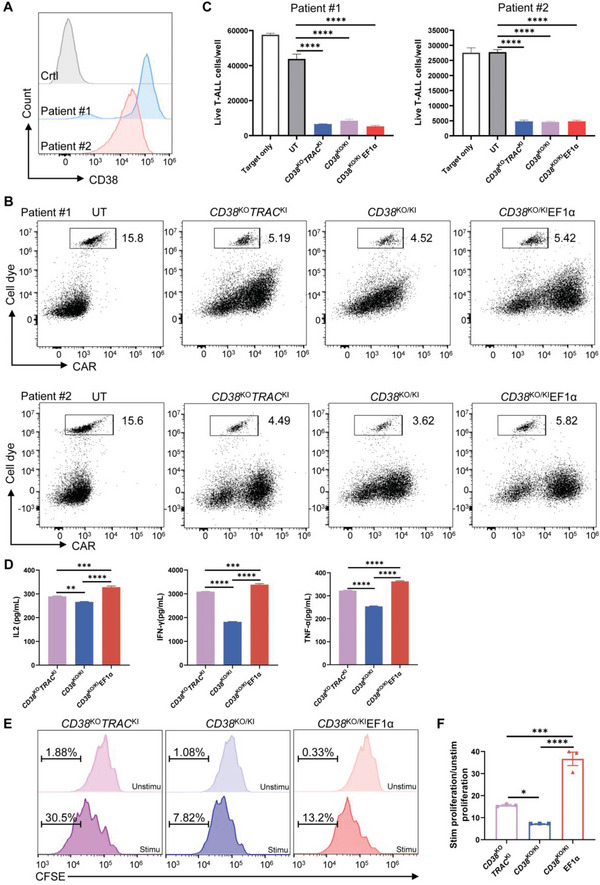
Antitumor activity of CD38 CAR T cells against primary T‐ALL cells. A) CD38 expression on blast cells of bone marrow samples from two T‐ALL patients, as measured by flow cytometry and presented as a representative histogram. B,C) Primary T‐ALL blast cells obtained from two patients were labeled with CytoTell Orange and co‐incubated at a 1:1 ratio with untransduced T (UT) or CD38 CAR‐T cells for 24h prior to FACS analysis. Counting beads were added to determine cell counts of viable primary T‐ALL cells. B) Dot plots show the percentage of live primary T‐ALL cells at the end of co‐culture. C) Absolute counts of live primary T‐ALL cells quantified by flow cytometry at the end of co‐culture. Data represent the mean ± SEM of triplicates. D) Concentrations of cytokine secreted by CAR‐T cells were detected after stimulation by primary T‐ALL cells. Data represent the mean ± SEM of triplicates. E) The proliferation of CAR‐T cells analyzed by CFSE assay. Unstim represents CAR‐T cells without stimulation and stim represents CAR‐T cells stimulated by primary T‐ALL cells for 72 h. F) CAR‐T cell antigen‐dependent proliferation fold calculated by stim/unstim ratio. Data represent the mean ± SEM of triplicates. *p < 0.05, **p < 0.01, ***p < 0.001, and ****p < 0.0001; ns, not significant.

In addition to the Jurkat cell line, we further tested the cytotoxicity of these CAR‐T cells against primary T‐ALL cells in vitro. We acquired two frozen bone marrow samples of T‐ALL patients whose blast cells expressed high levels of CD38 (**Figure** **4**A). Primary T‐ALL cells were labeled with dye to distinguish them from CAR‐T cells. After primary cells were incubated with CAR‐T cells, the residual viable T‐ALL cell numbers were quantified with Counting Beads. We observed robust cytotoxicity of CD38 CAR‐T cells against primary T‐ALL cells (Figure 4B), resulting in more than 70% elimination of T‐ALL cells for both patient samples (Figure 4C). After incubation with primary T‐ALL cells for 24 h, all three CAR‐T cells were observed to secrete cytokines, with *CD38* ^KO/KI^ CAR‐T cells secreting the lowest level of cytokines (Figure 4D). The baseline proliferation of the three CD38 CAR‐T cells was similar in the absence of antigen, and *CD38* ^KO/KI^ CAR‐T cells exhibited the lowest antigen‐dependent proliferation compared to *CD38* ^KO^ *TRAC* ^KI^ and *CD38* ^KO/KI^EF1α CAR‐T cells (Figure 4E,F), which is consistent with the results using Jurkat cells as target cells (Figure 3G–I). Therefore, these CD38 CAR‐T cells also demonstrated efficacy against primary T‐ALL cells in vitro.

### 2.5 *CD38* ^KO/KI^EF1α and *CD38* ^KO^ *TRAC* ^KI^ CAR‐T cells Showed Higher Antitumor Activity than *CD38* ^KO/KI^ CAR‐T Cells In Vivo

**Figure 5:**
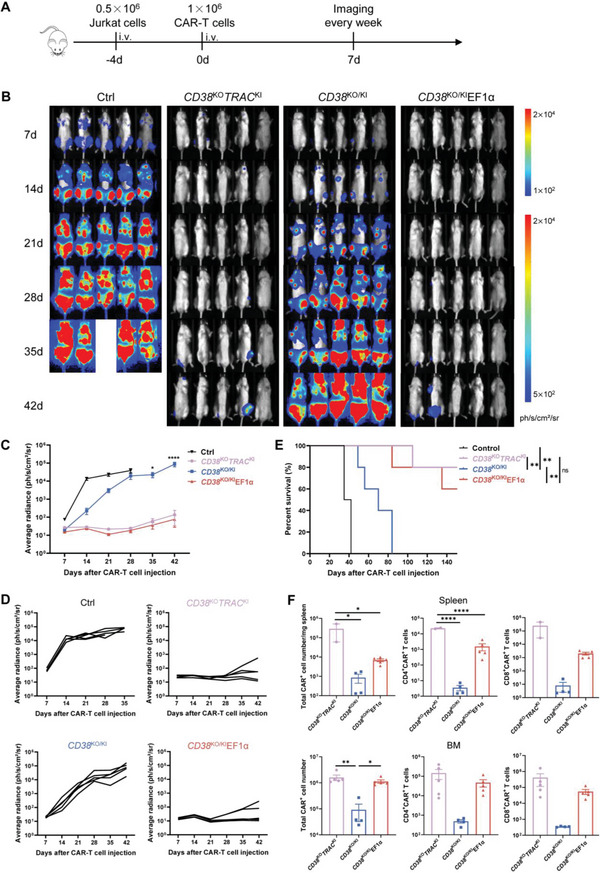
*CD38* ^KO/KI^EF1α and *CD38* ^KO^ *TRAC* ^KI^ CAR‐T cells exhibited better antitumor activity than *CD38* ^KO/KI^ CAR‐T cells in a mouse xenograft T‐ALL model. A) Experimental timeline. Mice (*n* = 5 per group) were injected intravenously with 0.5 × 10^6^ Jurkat‐FFLuc‐EGFP cells, followed by intravenous injection of 1 × 10^6^ of untransduced T or CD38 CAR T cells 4 days later, and the tumor burden was monitored by weekly imaging. B) Representative bioluminescence imaging (BLI) results of tumor burden are shown (donor 1). Ctrl, Control refers to untransduced T (UT) treated mice. C) Kinetics of tumor progression (average radiance) evaluated by bioluminescence imaging (donor 1). Data were shown as mean ± SEM. Statistical analysis was performed between *CD38* ^KO/KI^ and *CD38* ^KO/KI^EF1α groups. D) Tumor burden (average radiance) of mice in each group is shown (donor 1). E) Kaplan–Meier survival curve of mice treated with control or CD38 CAR‐T cells (donor 2). F) The numbers of CAR T cells in the bone marrow and spleen of mice 49 days post‐infusion (donor 1). Data were shown as mean ± SEM. *p < 0.05, **p < 0.01, ***p < 0.001, and ****p < 0.0001; ns, not significant.

Next, to evaluate the in vivo antitumor efficacy of these CAR‐T cells, we used a mouse xenograft model of T‐ALL in which Jurkat‐luciferase cells were intravenously injected into immunodeficient mice. Four days after tumor engraftment, we injected a low dose (1 × 10^6^) of CD38 CAR T cells and measured the tumor burden by weekly bioluminescence imaging (**Figure** **5**A). We observed much better tumor eradication in mice treated with *CD38* ^KO^TRAC^KI^ and *CD38* ^KO/KI^EF1α CAR‐T cells than in mice treated with *CD38* ^KO/KI^ CAR‐T cells (Figure 5B–D). This result was confirmed in an independent experiment with a second donor (Figure S4A,B, Supporting Information). Similarly, a significant improvement in overall survival was observed in mice receiving *CD38* ^KO^ *TRAC* ^KI^ and *CD38* ^KO/KI^EF1α CAR‐T cells, but *CD38* ^KO/KI^ cells only had a minor effect on overall survival (Figure 5E). We extracted the spleen and bone marrow from mice at day 49 and analyzed the phenotypes of CAR‐T cells (Figure S5, Supporting Information). This ex vivo analysis revealed that the number of *CD38* ^KO^ *TRAC* ^KI^ CAR‐T cells accumulated in both spleen and bone marrow was the highest, followed by *CD38* ^KO/KI^EF1α CAR‐T cells, while the number of *CD38* ^KO/KI^ CAR‐T cells was the lowest (Figure 5F). *CD38* ^KO/KI^ CAR‐T cells demonstrated a higher percentage of terminally differentiated CD62L^−^CD45RA^+^ effector cells (T_EFF_) in both the spleen and bone marrow. A nonsignificant but slightly higher proportion of PD‐1^+^LAG‐3^+^ CAR‐T cells was also detected in *CD38* ^KO/KI^ CAR‐T cells (Figure S4C, Supporting Information). Therefore, *CD38* ^KO^ *TRAC* ^KI^ and *CD38* ^KO/KI^EF1α CAR‐T cells demonstrated potent antitumor activity in vivo, while C*D38* ^KO/KI^ CAR‐T cells were not as effective at this low dose.

### 2.6 *CD38* ^KO/KI^ CAR‐NK Cells Displayed Superior Antitumor Effects In Vitro And In Vivo

**Figure 6:**
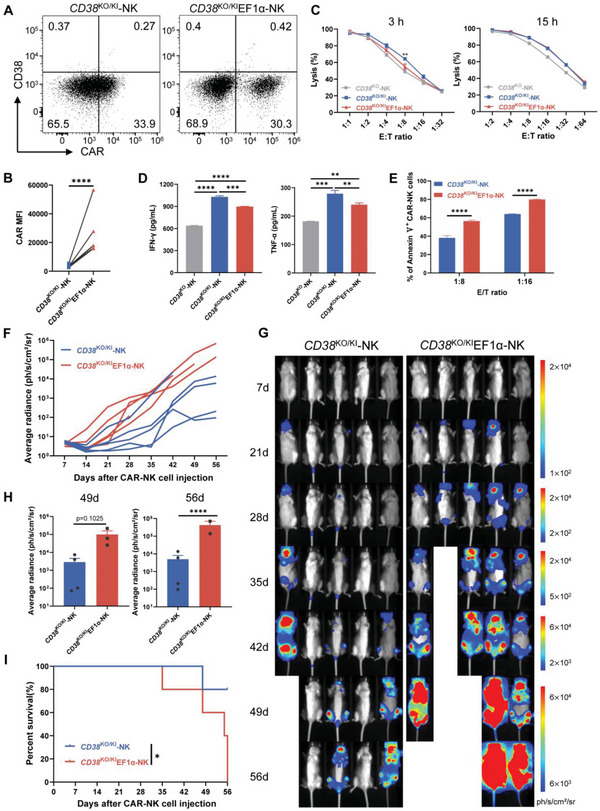
*CD38* ^KO/KI^ CAR‐NK cells displayed superior antitumor effects in vitro and in vivo. A) Flow cytometric analysis showing the expression of CD38 and CAR in NK cells 4 days after gene transduction. Data are representative of at least five independent experiments with similar results. B) CAR MFI 4 days after gene transduction (*n* = 6 donors). The paired comparison within each donor was connected by a line. C) Cytotoxic activity of CAR‐NK cells toward target cells as determined by a 3 h (left) and 15 h (right) bioluminescence assay, *CD38* ^KO^NK cells were used as the control. Data represent the mean ± SEM of triplicates from one of the three independent experiments. D) Cytokine secretion by CAR‐NK cells was detected after stimulation by target cells. Data represent the mean ± SEM of triplicates from one of the three independent experiments. E) Percentages of apoptotic CAR‐NK cells expressing Annexin V were determined. F–I) 6.5 × 10^6^ CD38 CAR‐NK cells derived from donor 1 were injected. F) Kinetics of tumor progression (average radiance) evaluated by bioluminescence imaging. G) Representative bioluminescence imaging (BLI) results of tumor burden are shown. H) The tumor burden (average radiance) of mice in each group at days 49 and 56 after treatment was shown. Data were shown as mean ± SEM. I) Kaplan–Meier survival curve of mice treated with CD38 CAR‐NK cells. *p < 0.05, **p < 0.01, ***p < 0.001, and ****p < 0.0001; ns, not significant.

Using CAR‐NK cells to treat T‐ALL can resolve the problem of malignant T cell contamination, but CD38‐specific CAR‐NK cells would also cause fratricide as they also express CD38. Thus, we employed the same “2‐in‐1” strategy to produce CD38 CAR‐NK cells (Figure 3A). Since NK cells do not express T‐cell receptor α, we omitted the *CD38* ^KO^ *TRAC* ^KI^ design and generated *CD38* ^KO/KI^ and *CD38* ^KO/KI^EF1α CAR‐NK cells. Targeting efficiencies of CARs were about 30% (**Figure** **6**A), and we observed uniform expression of *CD38* ^KO/KI^ CAR in multiple donors. In contrast, *CD38* ^KO/KI^EF1α CAR showed variegated expression with six‐fold higher MFI (Figure 6B).

We next determined the lytic capacity of the two CD38 CAR‐NK cells against Jurkat cells. Both CAR‐NK cells showed slightly higher cytotoxicity than control NK cells. And *CD38* ^KO/KI^ CAR‐NK cells were better at lysing target cells than *CD38* ^KO/KI^EF1α cells after they were co‐cultured with target cells for 3 h (Figure 6C). Both CD38 CAR‐NK cells produced more cytokines than *CD38* ^KO^ NK cells. Notably, *CD38* ^KO/KI^ CAR‐NK cells secreted significantly higher levels of IFN‐γ and TNF‐α than *CD38* ^KO/KI^EF1α cells (Figure 6D and Figure S6A, Supporting Information). After being stimulated with the target cells for 24 h, more *CD38* ^KO/KI^EF1α CAR‐NK cells showed apoptosis compared to *CD38* ^KO/KI^ CAR‐NK cells at different E:T ratios, indicating *CD38* ^KO/KI^ CAR‐NK cells had less activation‐induced cell death (AICD) (Figure 6E and Figure S6B, Supporting Information). To our surprise, *CD38* ^KO/KI^ CAR‐NK cells could eliminate tumors significantly better than *CD38* ^KO/KI^EF1α cells in the mouse T‐ALL model at day 56 in donor 1 and day 28 in donor 3 (Figure 6F–H and Figure S7, Supporting Information). A significantly improved overall survival was observed in mice receiving *CD38* ^KO/KI^ CAR‐NK cells (Figure 6I). We collected the peripheral blood of the mice on day 3 and 7 after CAR‐NK cell infusion and counted the absolute number of NK cells. There appeared to be slightly more NK cells in *CD38* ^KO/KI^ group than in *CD38* ^KO/KI^EF1α group at both time points, but there was no statistical difference due to high intragroup variance (Figure S8A, Supporting Information). We also extracted the spleen and bone marrow from treated mice at day 35 and tried to analyze the NK cells. Unfortunately, we could not detect any NK cells in both groups, probably because NK cells have a limited lifespan^[^ ^36^, ^37^, ^38^ ^]^ (Figure S8B, Supporting Information). The observation that *CD38* ^KO/KI^ CAR‐NK was better than *CD38* ^KO/KI^EF1α CAR‐NK in vitro and in vivo was completely opposite to the results in the CAR‐T scenario.

### 2.7 T and NK Cells have Distinct Expression Levels of CD38

**Figure 7:**
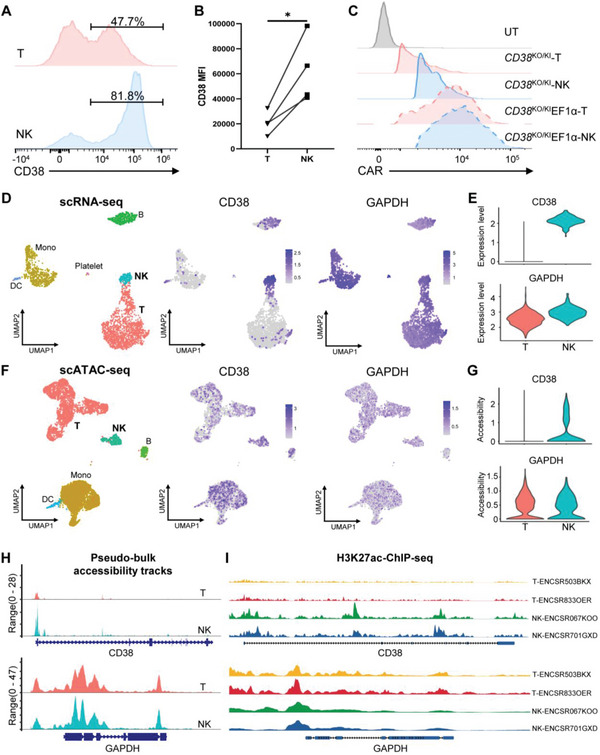
T and NK cells displayed distinct expression levels of CD38. A) CD38 expression of T and NK cells isolated from the peripheral blood without activation, as measured by flow cytometry and presented as a representative histogram. B) CD38 MFI of T and NK cells isolated from the peripheral blood without activation (*n* = 4 donors). C) CAR MFI of CAR‐T/NK cells generated by "2‐in‐1" strategy. D) Left, UMAP depicting cell type clustering analysis of single‐cell RNA‐seq dataset of human peripheral blood mononuclear cells (PBMCs). Middle and right, expression of CD38 and GAPDH (control) in PBMCs, respectively. E) Violin plot depicting the expression of *CD38* and *GAPDH* in PBMCs. A method based on low‐rank matrix approximation was used to impute the false zeros of gene expression. F) Left, UMAP depicting cell type clustering analysis of single‐cell ATAC‐seq dataset of PBMCs. Middle and right, accessibility of peaks around the transcription start site (TSS) of *CD38* and *GAPHD* genes in PBMCs, respectively. G) Violin plot depicting the accessibility of peaks around TSS of *CD38* and *GAPDH* in PBMCs. H) The pseudo‐bulk accessibility tracks depicting the frequency of Tn5 integration across regions, including 2kb up and downstream of *CD38* and *GAPDH* for cells grouped by cell type. Signals from all cells within a group have been averaged together to visualize the DNA accessibility in a region. I) Signal fold change over input control of H3K27ac ChIP‐seq in *CD38* locus and *GAPDH* locus. The datasets were downloaded from The Encyclopedia of DNA Elements (ENCODE) project (accession: ENCSR503BKX, ENCSR833OER, ENCSR067KOO, and ENCSR701GXD).

It is intriguing to see such differences between CAR‐T and CAR‐NK cells. A previous study suggested that NK cells express higher levels of CD38 than T cells.^[^ ^39^ ^]^ Indeed, when we analyzed CD38 protein levels in peripheral T and NK cells isolated from the same donor, we observed that NK had both a higher percentage of CD38^+^ cells and a higher CD38 MFI, which was confirmed in multiple donors (**Figure** **7**A,B and Figure S9, Supporting Information). In addition, the MFI of CD38 CAR expressed from the *CD38* locus of NK cells was about twofold that expressed from the same locus of T cells (Figure 7C). Through re‐analyzing human peripheral blood mononuclear cells (PBMCs) scRNA‐seq datasets in public databases, we confirmed that CD38 transcription is significantly higher in NK than in T cells (Figure 7D,E). Thus, the same *CD38* locus in NK and T cells was regulated differently, leading to higher expression of both endogenous CD38 and inserted exogenous CAR gene in NK cells. Indeed, re‐analyzing the single‐cell assay for transposase‐accessible chromatin with high‐throughput sequencing (scATAC‐seq) of PBMCs showed that the promoter of CD38 is more accessible in NK than T cells (Figure 7F–H). Moreover, analysis of H3K27ac chromatin immunoprecipitation assays with sequencing (ChIP‐seq) showed that NK cells had a higher level of H3K27ac than in T cells at the *CD38* locus while both cells had similar levels of H3K27ac at the control *GAPDH* locus (Figure 7I).

## 3 Discussion

Inspired by the success of CD38 monoclonal antibodies, many preclinical studies on CAR‐T and CAR‐NK cell therapies targeting CD38 have been conducted and shown promising results in the treatment of MM, AML, B‐ALL, and B‐NHL.^[^ ^28^, ^33^, ^40^, ^41^, ^42^, ^43^ ^]^ Clinical trials of CD38‐specific CAR‐T cells are in progress for the treatment of MM, AML, and B‐ALL. Our results presented here demonstrated the feasibility of CD38 CAR‐T/NK cell therapy against T‐ALL, expanding the application of CD38 CARs, which is also consistent with a recently published work.^[^ ^44^ ^]^ In our study, *CD38* ^KO/KI^EF1α CAR‐T cells efficiently eliminated T‐ALL cell lines and primary T‐ALL cells from patients in vitro and displayed potent antitumor activity in a mouse T‐ALL model. *CD38* ^KO/KI^ CAR‐NK cells could also efficiently kill T‐ALL cells in vitro and in vivo. These data paved the way for clinical trials of CD38‐specific CAR‐T/NK therapy to treat T‐ALL.

Our data confirmed extensive fratricide occurred with CD38 CAR‐T cells when the *CD38* gene was intact (Figure 1F,G). Utilizing CRISPR/Cas9 to disrupt *CD38* prevented fratricide and promoted the expansion of CD38 CAR‐T cells (Figures 2D and 3E), which was consistent with previous results of CD38 CAR‐NK cells.^[^ ^28^ ^]^ The gRNA targeting CD38 was from the study by Naeimi Kararoudi et al.,^[^ ^34^ ^]^ in which they surveilled the off‐target effects of the gRNA in edited NK cells and showed the specificity and safety of this gRNA.

Previously Eyquem et al. targeted a CD19‐specific CAR to the *TRAC* locus of T cells, with edited *TRAC* ^KI^CD19 CAR‐T cells showing lower CAR expression than retrovirally transduced CD19 CAR‐T cells, which reduced tonic signaling of CD19 CAR‐T cells.^[^ ^35^ ^]^ Similarly, *CD38* ^KO^ *TRAC* ^KI^ CAR‐T cells exhibited a lower CAR expression level than *CD38* ^KO^RV CAR‐T cells in our study (Figure 2C). And the evidence of more *CD38* ^KO^RV CAR‐T cells with phosphorylated CD3ζ than *CD38* ^KO^ *TRAC* ^KI^ CAR‐T cells in the absence of antigen stimulation indicated that *CD38* ^KO^RV CAR‐T cells have higher tonic signaling (Figure S2A, Supporting Information). Moreover, one study has shown that GD2.28z CAR‐T cells with higher tonic signaling expanded less efficiently than CD19.28z CAR‐T cells with lower tonic signaling in vitro,^[^ ^45^ ^]^ suggesting the correlation between higher tonic signaling and lower expansion capacity. Therefore, the lower CAR expression level and tonic signaling in *CD38* ^KO^ *TRAC* ^KI^ CAR‐T cells may explain why *CD38* ^KO^ *TRAC* ^KI^ CAR‐T cells showed better in vitro expansion than *CD38* ^KO^RV CAR‐T cells (Figure 2D and Figure S2B, Supporting Information). What is more, Eyquem et al. demonstrated that the edited *TRAC* ^KI^CD19 CAR‐T cells displayed superior antitumor effects than retrovirally transduced CD19 CAR‐T cells in a mouse B‐ALL model. Therefore, targeting a CAR to the *TRAC* locus by CRISPR/Cas9 may be a generalizable method to enhance the therapeutic potency of an existing CAR‐T product generated by retroviruses.

Our data showed that *CD38* ^KO^RV CAR‐T cells had a 30% higher TIM‐3 expression level than *CD38* ^KO^ *TRAC* ^KI^ CAR‐T cells when stimulated by target cells three times (Figure 2J and Figure S2I, Supporting Information). In general, TIM‐3 serves as an exhaustion marker of the dysfunctional T cell in cancer, which usually co‐expresses with PD1. But it may not only act as a marker. It is known that the binding of TIM‐3 with its ligand will result in the suppression of TCR signaling.^[^ ^46^ ^]^ In theory, a lower TIM‐3 expression level would reduce suppression of TCR signaling and result in higher T cell functions. One study has reported that TIM‐3 knockdown in vivo significantly increased IFN‐γ production from hepatic CD8^+^ T cells in the Hepatitis B virus mouse model.^[^ ^47^ ^]^ In addition, another research studied the effect of TIM‐3 knockout or TIM‐3 overexpression on T cells and confirmed the negative effect of TIM‐3 on T cell responses against acute lymphoblastic leukemia.^[^ ^48^ ^]^ Therefore, the difference in TIM‐3 expression level may be partly responsible for the functional discrepancy of *CD38* ^KO^RV and *CD38* ^KO^ *TRAC* ^KI^ CAR‐T cells.

Simultaneous targeting of two genes in *CD38* ^KO^ *TRAC* ^KI^ CAR‐T cells increases the off‐target risk and the experimental costs, which highlights the need for a simplified combinatorial KO/KI gene targeting strategy. In our study, *CD38* had to be knocked out to reduce fratricide. Meanwhile, an insertion site for targeted gene delivery could be created at the disrupted *CD38* locus by Cas9/gRNA RNP. So, we designed a “2‐in‐1” strategy to insert the CD38 CAR gene into the recycled *CD38* locus and placed the CD38 CAR gene under the transcriptional control of either the endogenous *CD38* promoter or an exogenous EF1α promoter to investigate the effect of CAR expression level on the function of CD38 CAR‐T/NK cells (Figure 3A). *CD38* ^KO/KI^EF1α CAR showed higher MFI than *CD38* ^KO/KI^ CAR in both T and NK cells, and *CD38* ^KO/KI^ CAR expression was more consistent than *CD38* ^KO/KI^EF1α CAR in both T and NK cells (Figures 3C and 6B). In the case of T cells, Eyquem et al. reported that CD19 CARs driven by EF1α promoter at the *TRAC* locus showed higher CAR MFI than CD19 CARs driven by *TRAC* promoter,^[^ ^35^ ^]^ but CD38 CARs under the control of EF1α promoter at the *CD38* locus of T cells showed a similar CAR MFI to CD38 CARs driven by *TRAC* promoter at the *TRAC* locus (Figure 3C). All of the observations suggested that the expression level of CARs is not only controlled by the promoter but also affected by the locus.

In T cells, *CD38* ^KO/KI^EF1α CAR‐T cells generated through our “2‐in‐1” strategy showed similar antitumor activity in vivo to *CD38* ^KO^ *TRAC* ^KI^ CAR‐T cells (Figure 5B–E). However, the production of *CD38* ^KO^ *TRAC* ^KI^ CAR‐T cells requires one additional type of gRNA and larger amounts of RNPs than *CD38* ^KO/KI^EF1α CAR‐T cells, which may increase the “off‐target” risk and result in the poor expansion of CAR‐T cells (Figure S3A, Supporting Information). Thus, *CD38* ^KO/KI^EF1α CAR‐T cells generated through our strategy have more advantages for autologous CD38 CAR‐T therapy than *CD38* ^KO^ *TRAC* ^KI^ CAR‐T cells. On the other hand, when allogeneic T cells were used to generate universal CD38 CAR‐T cells, *CD38* ^KO^ *TRAC* ^KI^ CAR‐T cells may be a better choice because TCR needs to be disrupted to prevent GvHD regardless.

CAR‐T cell therapy targeting T cell malignancies requires the separation of healthy T cells from malignant ones to avoid product contamination. We used NK cells to address this challenge. *CD38* ^KO/KI^ CAR‐NK cells generated through our strategy exhibited robust tumor control capability. Importantly, both allogeneic and autologous NK cells can be used to generate *CD38* ^KO/KI^ CAR‐NK cells for therapy. Taken together, our results showed the efficiency of our “2‐in‐1” strategy in both T and NK cells against T‐ALL. For other malignancies with high CD38 expression, such as MM and AML, CAR‐T/NK cells by the “2‐in‐1” strategy are likely to be effective as well, but further experiments are needed to prove that.

Intriguingly, *CD38* ^KO/KI^EF1α CAR‐T showed better antitumor activity than *CD38* ^KO/KI^ CAR‐T, while *CD38* ^KO/KI^ CAR‐NK displayed better tumor control than *CD38* ^KO/KI^EF1α CAR‐NK (Figure S10, Supporting Information). So, we did some preliminary experiments to explore what contributed to this difference. We found that the protein level of CD38 on the surface of freshly isolated peripheral NK was higher than that of T cells from the same donor, and this was corroborated by scRNA‐seq analysis (Figure 7A,B,D,E). Moreover, NK cells exhibited higher chromatin accessibility and H3K27ac level around the *CD38* locus than T cells (Figure 7F–I). The expression level of CARs inserted at the *CD38* locus was higher in NK cells than in T cells as well (Figure 7C). We speculated that the expression of CD38 CARs regulated by CD38 intrinsic promoters in T cells is too low that *CD38* ^KO/KI^ CAR‐T cells could not be activated efficiently after being stimulated by antigen, resulting in poor antitumor effect in vitro and in vivo. Conversely, CD38 CARs driven by *CD38* intrinsic promoters of NK cells display higher expression, allowing *CD38* ^KO/KI^ CAR‐NK cells to be better activated after stimulation. However, compared to *CD38* ^KO/KI^EF1α CAR‐T cells, CAR expression in *CD38* ^KO/KI^EF1α CAR‐NK cells was even higher, which may lead to more AICD of *CD38* ^KO/KI^EF1α CAR‐NK cells (Figure 6E and Figure S6B, Supporting Information), causing less effective antitumor activity in vivo. Therefore, our results suggested that the optimal "2‐in‐1" promoter design to achieve potent antitumor activity was not the same for T and NK cells.

In summary, our study demonstrated the feasibility of CD38 CAR for targeting T‐ALL. The “2‐in‐1” KO/KI strategy enabled knockout of the target gene and knockin of the CAR gene at the same locus, resolving fratricide. We also demonstrated CD38 CAR‐T and CAR‐NK cells generated by this strategy efficiently eliminated tumors in vitro and in vivo. Furthermore, the different antitumor activities caused by *CD38* ^KO/KI^ CAR and *CD38* ^KO/KI^EF1α CAR in T and NK cells suggested that transcriptional regulation of CAR expression is critical for effective tumor eradication, and the optimal CAR design may be different for T and NK cells. In addition to CD38, our “2‐in‐1” strategy should be applicable to CAR therapies that target other T‐lineage antigens such as CD5 and CD7, and transcriptional regulation of CAR expression should also be carefully accessed when designing such CARs.

## 4 Experimental Section

### Cell Lines

Jurkat cells (T‐ALL cell line) were lentivirally transduced to express FFLuc‐EGFP, and EGFP‐positive cells were sorted by flow cytometry. 293T cells were cultured in Dulbecco's modified Eagle medium (DMEM) (Gibco) with 10% fetal bovine serum (FBS) (Vistech) and 1% penicillin/streptomycin (Gibco). Jurkat cells were cultured in a complete RPMI‐1640 medium (Gibco) with 10% FBS and 1% penicillin/streptomycin.

### CAR Design and Constructs

The coding sequences of three CD38‐specific scFvs derived from 056, Ab79, and 3079 clones of CD38‐specific antibodies were synthesized commercially (GenScript). These scFvs were cloned into the backbone of a second‐generation CAR containing CD28 and CD3ζ domains. For CAR cloned into retroviral SFG vectors,^[^ ^35^, ^49^ ^]^ the CAR construct was modified to express EGFP via a P2A peptide as an alternative maker of CAR expression; and for CAR cloned into pAAV vector,^[^ ^35^, ^49^ ^]^ an HA tag was attached after CD8a leader signal peptide to enable the detection of CAR. The detailed methods of cloning were as previously described.^[^ ^50^ ^]^

### Isolation and Expansion of Human T Cells

Peripheral blood was obtained from healthy volunteers. Ethical permission was granted by the School of Medicine, Zhejiang University, and all of the blood samples were handled following the required ethical and safety procedures. PBMCs were isolated by Ficoll density gradient centrifugation (Dakewe). Then T lymphocytes were purified using the Pan T Cell Isolation Kit (Miltenyi Biotec) and activated with CD3/CD28 T cell Activator Dynabeads (Thermo Fisher Scientific) and cultured in X‐VIVO15 Serum‐free Hematopoietic Cell Medium (Lonza) supplemented with 10% FBS, 1% penicillin/streptomycin, 5 ng ml^−1^ interleukin‐7 (IL7) and 5 ng ml^−1^ interleukin‐15 (IL15) (Novoprotein). The medium was changed every 2 days, and cells were plated at 10^6^ cells ml^−1^.

### Isolation and Culture of Human NK Cells

NK cells were isolated from PBMCs using a CD56^+^ selection kit (Miltenyi Biotec) and then activated with irradiated K562 feeder cells (with surface expression of IL21 and 4‐1BBL) at a ratio of 1:2 in RPMI1640 medium supplemented with 10% FBS, 1% penicillin/streptomycin, 1% L‐glutamine (Gibco), 5 ng ml^−1^ IL15, and 200 U ml^−1^ IL‐2 (Novoprotein). The cells were cultured at 37 °C and 5% CO_2_ and changed into fresh medium 3 days after isolation.

### Guide RNA

*TRAC* gRNA sequence:

5′‐C*A*G*GGUUCUGGAUAUCUGUGUUUUAGAGCUAGAAAUAGCAAGUU
AAAAUAAGGCUAGUCCGUUAUCAACUUGAAAAAGUGGCACCGAGUCGGUGCU*U*U*U‐3′.

*CD38* gRNA sequence:

5′‐C*T*G*AACTCGCAGTTGGCCATGUUUUAGAGCUAGAAAUAGCAAGUU
AAAAUAAGGCUAGUCCGUUAUCAACUUGAAAAAGUGGCACCGAGUCGGUGCU*U*U*U‐3′. Asterisk (*) represents 2′ ‐O‐methyl 3′ phosphorothioate.

### Ribonucleoprotein (RNP) Production

RNPs were produced by complexing gRNA and Cas9 protein. Modified gRNAs were synthesized by GeneScript and reconstituted at 1 µg µl^−1^ in RNase‐free water. Cas9 proteins were produced by the lab as described in the previous work.^[^ ^51^ ^]^ In brief, they were complexed in a 2:1 gRNA‐to‐Cas9 molar ratio at room temperature for 20 min and then electroporated into T cells immediately after complexing.

### Gene Targeting of T/NK Cells and Production of CAR‐T/NK Cells

CD3/CD28 beads were removed magnetically 48 h after initiating T cell activation, and T cells were transfected by electroporation of RNPs complex for the production of CAR‐T cells; three days after stimulation by feeder cells, NK cells were also electroporated with RNPs complex for the production of CAR‐NK cells. Briefly, for targeting the *CD38* gene only, 1 × 10^6^ cells were mixed with 80 pmol Cas9‐CD38 gRNA RNPs into a 120 ul cuvette and electroporated with a cell electroporator (CELETRIX); for targeting the *CD38* gene and *TRAC* gene simultaneously, 80 pmol Cas9‐CD38 gRNA RNPs and 120 pmol Cas9‐TRAC gRNA RNPs were needed for 1 × 10^6^ cells. Cells were transferred into the culture medium after electroporation. For AAV transduction, a recombinant AAV6 donor vector (Vigene Biosciences) was added to the culture medium at the indicated multiplicity of infection 30 min after electroporation. For retroviral transduction, T cells were transferred to Retronectin (Takara)‐coated plates with medium without cytokines 24 h after electroporation, and transduction was performed by addition of retrovirus followed by centrifugation. Cells were changed to a fresh complete medium with cytokines 24 h after transduction. Four days after gene targeting, some cells were harvested for FACS analysis to determine the transduction efficiency.

### Cytotoxicity Assays

The cytotoxicity of CAR‐T and CAR‐NK cells was determined by standard luciferase‐based assays. In brief, Jurkat cells expressing FFLuc‐EGFP served as target cells. The effector (E) and tumor target (T) cells were co‐cultured in triplicates at the indicated E/T ratio using black‐walled 96‐well plates with 5 × 10^4^ target cells in a total volume of 100 µl per well in RPMI medium. Target cells alone were plated at the same cell density to determine the maximal luciferase expression (relative light units (RLU)); 18 h later, 100 µl of 150 ng ml^−1^ luciferase substrate (Goldbio) was directly added to each well. Emitted light was detected in a luminescence plate reader. Lysis was determined as (1‐(RLUsample)/(RLUmax)) × 100.

### Primary T‐ALL Cells

Bone marrow samples were obtained from acute T‐cell leukemia patients through bone marrow aspiration, and mononuclear cells were isolated through density gradient centrifugation and stored frozen in liquid nitrogen. Frozen mononuclear cells from patients were thawed and directly used for cytotoxicity assays. All primary samples were obtained from The Children's Hospital, Zhejiang University, after informed consent and approval by the institutional medical ethical committee (2021‐IRB‐257).

### Flow Cytometry‐Based Cytotoxicity Assay

To determine the killing of primary T‐ALL cells by CAR‐T cells, primary T‐ALL cells were labeled with CytoTell Orange (AAT Bioquest) to distinguish primary tumor cells from CAR‐T cells. Labeled cells were co‐cultured with CAR‐T cells and untransduced T cells at a 1:1 ratio for 24 h, cells in each well were collected and stained with specific antibodies for flow cytometry. Dead cells were excluded by DAPI‐staining and absolute cell counts of viable target cells were quantified using CountBright Plus Absolute Counting Beads (Invitrogen).

### Cytokine Measurements

To determine cytokine production, CAR‐T/NK cells were co‐cultured with target cells at an E:T ratio of 4:1 for 24 h, and the cell culture supernatant was harvested. To measure cytokines, the Cytokine Bead Array (CBA) and Human Soluble Protein Master Buffer Kit (BD) were used according to the manufacturer's protocol. In brief, a mixture of capture beads (IL2, TNF‐α, and IFN‐γ) and cell supernatant was incubated for 1 h, then PE‐detection reagent was added to incubate for an additional 2 h. Beads were washed and analyzed by flow cytometry.

### CFSE Assays

For the proliferation assay, CAR‐T cells were labeled with CFSE. Briefly, CAR‐T cells were resuspended in RPMI 1640 with 10% FBS and 1% penicillin/streptomycin at a final concentration of 5 × 10^6^ cells per milliliter, then CFSE solution was added at the suggested working concentration. After that, CAR‐T cells were incubated at 37 °C for 10 min and washed three times with RPMI 1640 with 10% FBS and 1% penicillin/streptomycin. Labeled CAR‐T cells were stimulated with target cells in triplicates at a ratio of 4:1, and labeled CAR‐T cells without stimulation served as the control. Cytokines were added to the assay (5 ng ml^−1^ IL7 and 5 ng ml^−1^ IL15). After 72 h, cells in each well were collected and stained with indicated antibodies for flow cytometry.

### Antigen Stimulation and Proliferation Assay

For weekly antigen stimulation of CAR‐T cells, 5 × 10^5^ CAR‐T cells were co‐cultured with target cells at a ratio of 4:1 in culture medium with 5 ng ml^−1^ IL7 and 5 ng ml^−1^ IL15 in 24‐well plates. Total cells were counted and CAR expression was determined weekly by flow cytometry. Subsequently, CAR‐T cells were restimulated under the same conditions. For repeated proximal stimulation of CAR‐T cells, target cells were added to the culture medium containing CAR‐T cells every 24 h for once, twice, or thrice. CAR‐T cells were analyzed by FACS for the expression of CAR and exhaustion markers.

### Apoptosis Assay of CAR‐NK Cells

CAR‐NK cells were co‐cultured with target cells at different ratios in 24‐well plates with medium supplemented with 200 U ml^−1^ IL2 for 24 h, and then cells were collected and stained with the CAR and apoptotic marker Annexin V (YEASEN) and then analyzed by flow cytometry.

### Flow Cytometry

The following fluorophore‐conjugated antibodies were used.

From BD Biosciences: PE Mouse Anti‐Human CD38; PE Mouse Anti‐Human CD3; BV510 Mouse Anti‐Human CD279 (PD‐1); BV421 Mouse Anti‐Human CD62L; APC‐Cy7 Mouse Anti‐Human CD8; BUV395 Mouse Anti‐Human CD4; Alexa Fluor 488 Mouse Anti‐CD247 (pY142).

From BioLegend: Alexa Fluor 647 anti‐HA.11.

From Invitrogen: Anti‐Hu CD223 (LAG‐3), eFluor450; Anti‐Hu CD8a, PE; Anti‐Hu CD62L (L‐Selectin), eFluor450; Anti‐Hu CD45RA, eFluor506; Anti‐Hu CD223 (LAG‐3), PerCP‐eFluor710 (3DS223H); Anti‐Hu CD45RA, FITC (HI 100). DAPI and 7‐AAD (YESEN) were used as viability dyes.

For the detection of intracellular phosphorylated CD3ζ, CAR‐T cells were first fixed and permeabilized and then stained with the CAR and anti‐CD247 (pY142). For ex vivo experiments, Fc receptors were blocked using anti‐mouse CD16/CD32 (BioLegend). For cell counting, CountBright Absolute Counting Beads were added (Invitrogen). Flow cytometry assays were performed on Cytoflex (Beckman Coulter) and a FACS AriaIIsorter (BD Biosciences) was used for cell sorting. Data were analyzed with the FlowJo software v.10.1.

### In Vivo Xenograft Mouse Model

6‐ to 12‐week‐old NPSG (Jihui) mice were inoculated with 0.5 × 10^6^ Jurkat‐FFLuc‐EGFP cells by tail vein injection, and CD38 CAR‐T cells (1 × 10^6^) or CD38 CAR‐NK cells (dose depends on the donor) were injected 4 days later. To measure luminescence, mice were injected with D‐luciferin intraperitoneally. Tumor burden was monitored by a Biospace Optima small animal imaging system (Biospace Lab), and M3 vision software was used to visualize and calculate total luminescence. For ex vivo phenotype analysis of CAR‐T cells or CAR‐NK cells in BM and spleen, BM and spleen were collected from mice. After grinding, dissociated tissues were filtered through a 70 µm filter, and the single cell suspension was stained with antibodies for subsequent flow cytometric analysis. For ex vivo analysis of CAR‐NK cells in peripheral blood, blood from mice was collected, red blood cells were lysed, and then the single‐cell suspension was stained with antibodies for subsequent flow cytometric analysis. All of the mice were housed at Westlake University under pathogen‐free conditions, and the procedures were approved by the ethics committee of Westlake University (19‐039‐GXF).

### scRNA‐Analysis

Raw reads counts matrix was freely available from 10X Genomics and was processed using the Seurat package (v.3.1.4)21 in R (v.3.6.3).^[^ ^52^ ^]^ Low‐quality cells or doublets with fewer than 200 genes were detected, greater than 2500 genes were detected, or more than 5% mitochondrial reads were removed. SCTransfrom function was used to normalize and scale count matrix. Next, PCA was run, and then UMAP was performed using the first 20 principal components. Cells were clustered using Louvain at resolution 0.5. RunALRA function based on low‐rank matrix approximation was used to impute the false zeros of gene expression.^[^ ^53^ ^]^ Plots were generated using DimPlot, FeaturePlot, and VlnPlot with default parameters.

### scATAC‐Seq Analysis

Raw reads, counts matrix, and fragment file, including all unique fragments across all single cells, were freely available from 10× Genomics and were processed using the Signac package.^[^ ^54^ ^]^ Low‐quality cells or cells with transcription start site enrichment scores lower than 2, with fewer than 200 or greater than 2500 genes fragments in peaks, and DNA fragment sizes without nucleosome banding pattern (nucleosome signal > 4) were removed. Then, term frequency‐inverse document frequency (TF‐IDF) normalization using the RunTFIDF function was performed. The first LSI component often captured sequencing depth (technical variation) rather than biological variation, so the correlation between each LSI component and sequencing depth was assessed using the DepthCor function. SVD was run, and then UMAP was performed using the second to the thirtieth principal components. Plots were generated using DimPlot, FeaturePlot, and VlnPlot functions in Seurat^[^ ^52^ ^]^ and the CoveragePlot function in Signac^[^ ^54^ ^]^ with default parameters.

### H3K27ac ChIP‐seq Analysis

BigWig files were downloaded from The Encyclopedia of DNA Elements (ENCODE) project (ENCSR503BKX, ENCSR833OER, ENCSR067KOO, and ENCSR701GXD). The make_tracks_file and pyGenomeTracks functions in PyGenomeTracks packages^[^ ^55^ ^]^ were used to generate a configuration file and plot genomic tracks on specified regions, respectively.

### Statistical Analysis

All statistical analyses were performed using GraphPad Prism software version 8.0. Statistical comparisons between the two groups were determined by the Student *t*‐test. For three or more groups, one‐way analysis of variance (ANOVA) was used. A *p*‐value < 0.05 was considered significant.

### Ethics Approval and Consent to Participate

All animal studies were reviewed and approved by the Ethical Committee of Westlake University.

## Conflict of Interest

The authors declare no conflict of interest.

## Author Contributions

C.L., Y.W., and Y.H. contributed equally to the work. C.L. and Y.W. planned and performed experiments, analyzed the data, and wrote the paper; Y.H. planned and performed experiments; Y.D. performed experiments and analyzed data; J.C., J.J., and K.S. performed experiments; N.L. and Y.L. analyzed and interpreted data; C.Z. and Y.G. wrote the paper; X.Z., X.G., Y.T., and J.S. directed the study, planned the experiments, and wrote the paper.

## Supporting information

Supporting Information

## Data Availability

The data that support the findings of this study are available from the corresponding author upon reasonable request.
