# Supplementary material for: CD38‐Specific CAR Integrated into CD38 Locus Driven by Different Promoters Causes Distinct Antitumor Activities of T and NK Cells
Source: Adv Sci (Weinh). 2023 Jul 23;10(27):2207394. doi: 10.1002/advs.202207394 (PMC10520621; doi:10.1002/advs.202207394)
Supplement: Supplementary file 1 — Supporting Information [file ADVS-10-2207394-s001.pdf]

ADVANCED SCIENCE
Open Access

Supporting Information

for *Adv. Sci.*, DOI 10.1002/advs.202207394

CD38-Specific CAR Integrated into *CD38* Locus Driven by Different Promoters Causes Distinct Antitumor Activities of T and NK Cells

Chan Liao, Yajie Wang, Yanjie Huang, Yanting Duan, Yan Liang, Jiangqing Chen, Jie Jiang, Kai Shang, Chun Zhou, Ying Gu, Nan Liu, Xun Zeng, Xiaofei Gao*, Yongmin Tang* and Jie Sun**

Supporting information for

CD38-specific CAR integrated into *CD38* locus driven by different promoters causes distinct antitumor activities of T and NK cells

Chan Liao, Yajie Wang, Yanjie Huang, Yanting Duan, Yan Liang, Jiangqing Chen, Jie Jiang, Kai Shang, Nan Liu, Chun Zhou, Ying Gu, Xun Zeng, Xiaofei Gao*, Yongming Tang*, Jie Sun**

*Corresponding author: Jie Sun sunj4@zju.edu.cn; Yongming Tang y_m_tang@zju.edu.cn; Xiaofei Gao gaoxiaofei@westlake.edu.cn; Xun Zeng xunzeng@zju.edu.cn

Chan Liao, Yajie Wang and Yanjie Huang contributed equally to this work.

The PDF file includes:

Figure S1 to S10
Table S1

Supplementary Figures

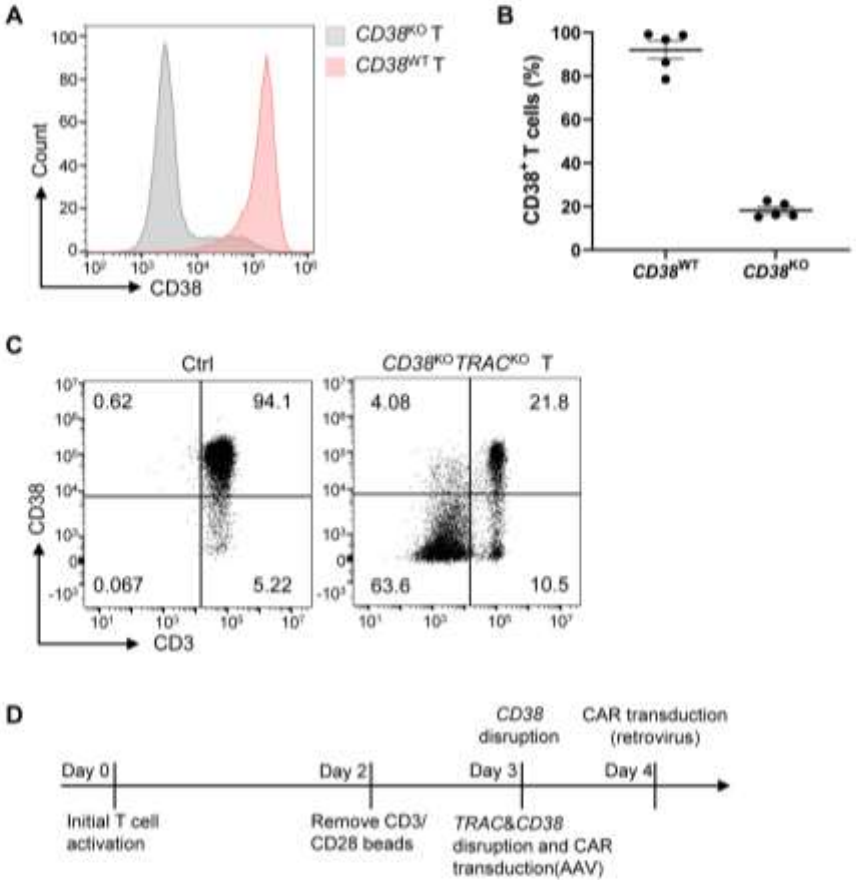

Figure S1: Efficient knockout of *CD38* and *TRAC* genes by CRISPR/Cas9. (A) Representative histograms of relative expression of CD38 on *CD38*^{WT} and *CD38*^{KO} T cells from one donor. KO, Knockout; WT, Wild type. **(B)** Pooled data showing the proportion of CD38-positive cells in *CD38*^{WT} and *CD38*^{KO} T cells (n=5 donors). **(C)** Dot plots showing expression of CD38 and CD3 on T cells 3 days after electroporation with Cas9/CD38 gRNA RNPs and Cas9/TRAC gRNA RNPs. Ctrl refers to unedited T cells; gRNA, guide RNA. **(D)** Schematic outline of the optimized protocol for generating *CD38*^{KO}*TRAC*^{KI} and *CD38*^{KO}RV CAR-T cells.

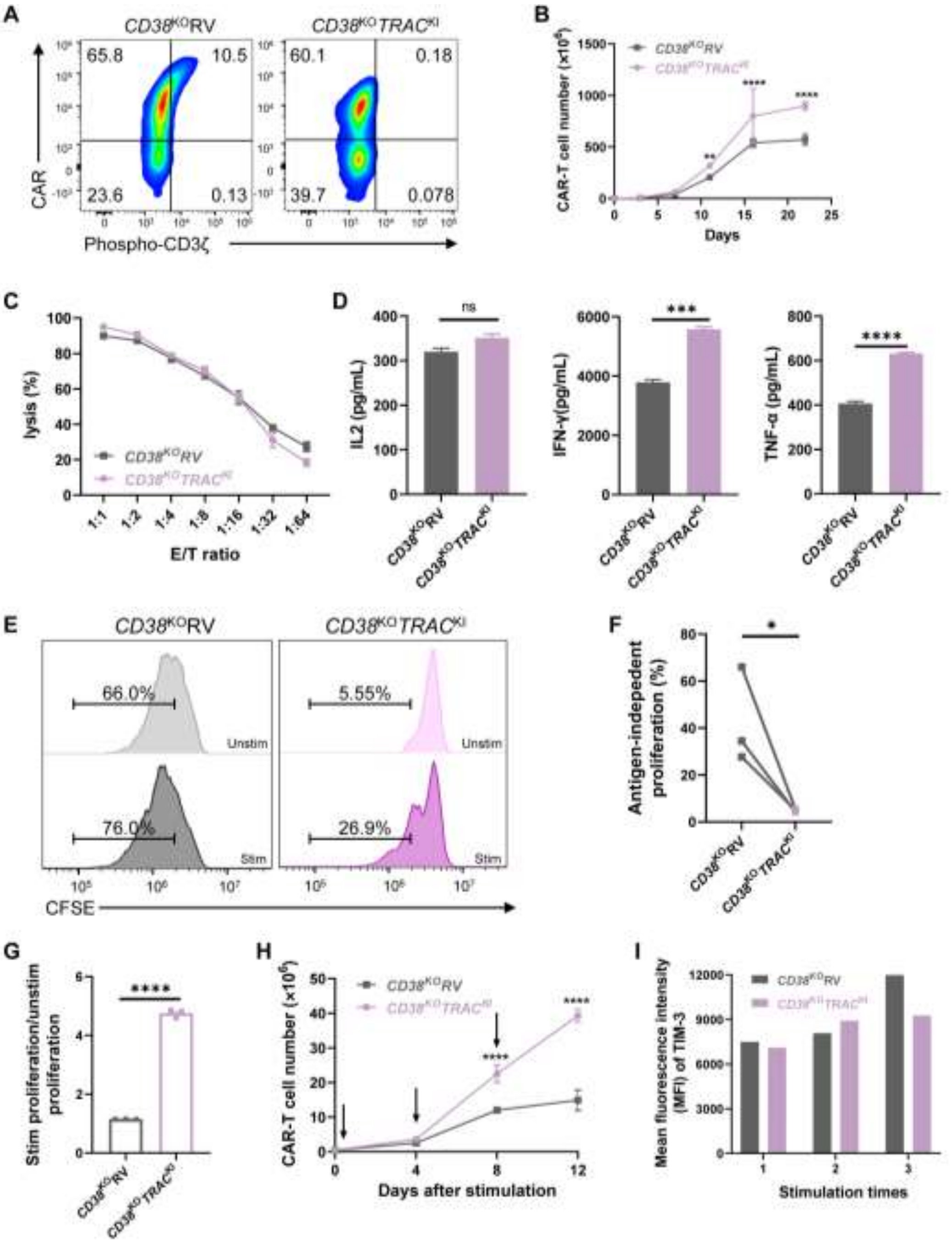

Figure S2: ***In vitro*** **functional assays of** ***CD38***$^{\mathbf{KO}}$***TRAC***$^{\mathbf{KI}}$ **and** ***CD38***$^{\mathbf{KO}}$**RV CAR T cells against target cells.** CAR-T cells in **(B-E)** and **(G-I)** were derived from Donor 2. **(A)** FACS analysis of CD3ζ phosphorylation of *CD38*^{KO}*TRAC*^{KI} and *CD38*^{KO}RV CAR T cells in the absence of antigen stimulation. **(B)** Expansion of CAR-T cells after transduction with CD38 CAR *in vitro.* **(C)** The cytotoxic activity of CAR T cells upon coculture with target cells. Data represent the mean ± SEM of triplicates. **(D)** Concentrations of cytokine secreted by both CAR-T cells. Data represent the mean ± SEM of triplicates. **(E)** The proliferation of CAR-T cells analyzed by CFSE assay. Unstim represents CAR-T cells without stimulation, stim

represents CAR-T cells stimulated by Jurkat cells for 72h. **(F)** The antigen-independent proliferation of both CAR-T cells (n=3 donors). The paired comparison within each donor was connected by a line. **(G)** CAR-T cell antigen-dependent proliferation fold calculated by stim/unstim ratio. Data represent the mean ± SEM of triplicates. **(H)** Cumulative cell numbers of CAR T cells upon continuous stimulations with target cells. **(I)** Quantification of TIM-3 expression levels of *CD38*^{KO}*TRAC*^{KI} CAR-T cells and *CD38*^{KO}RV CAR-T cells stimulated 1, 2 or 3 times by target cells in Figure 2J. Arrows indicate stimulation time points. Data represent the mean ± SEM of triplicates. *P < 0.05, **P < 0.01, ***P < 0.001, and ****P < 0.0001; ns, not significant.

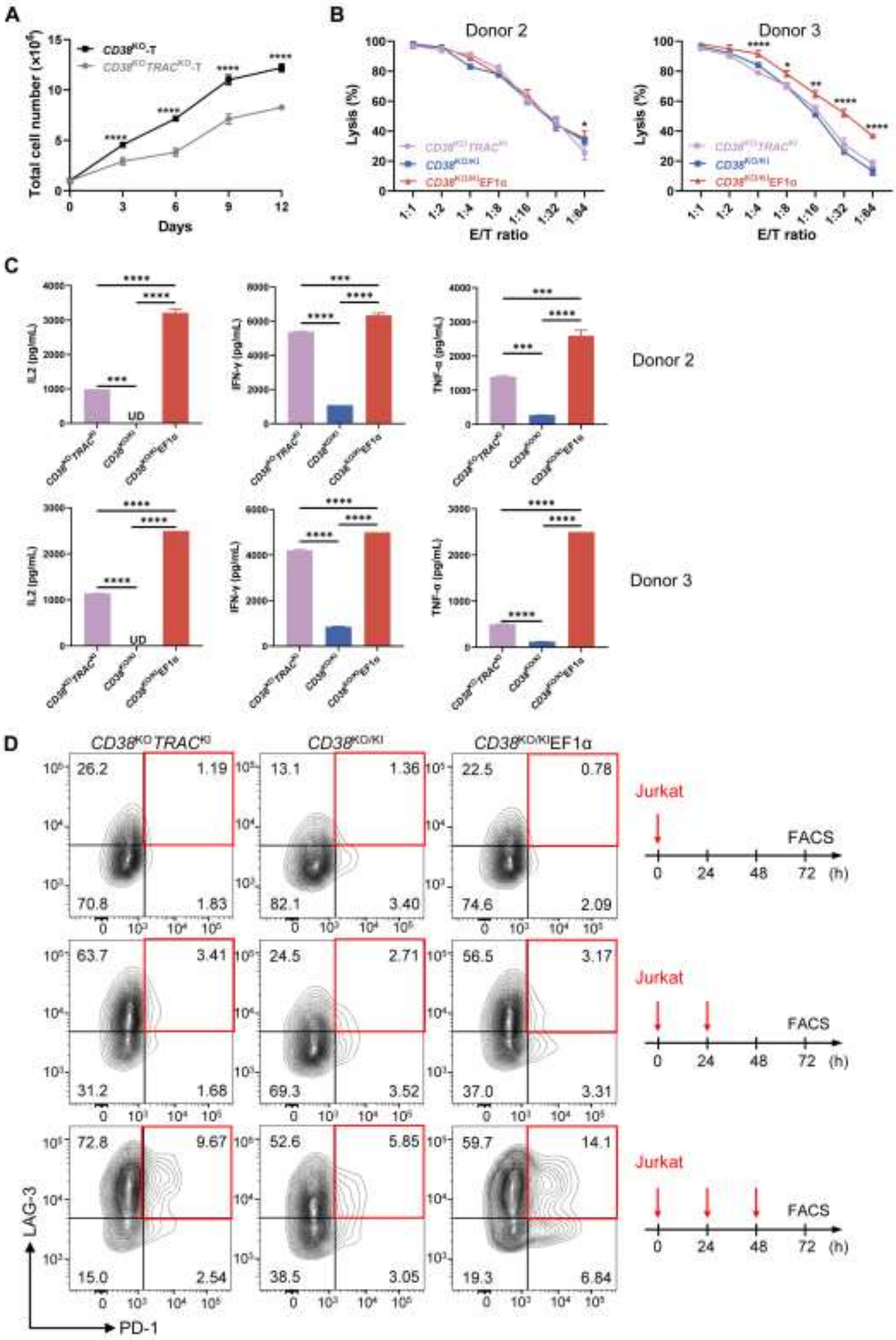

Figure S3: ***In vitro*** **functional assays of CD38 CAR-T cells generated by the "2-in-1" KO/KI strategies.** **(A)** Expansion of *CD38*^{KO} T cells and *CD38*^{KO}*TRAC*^{KO} T cells after gene editing. Cell numbers were counted every 3 days. **(B)** Cytotoxic activity of CAR-T cells from two additional donors against target cells using an 18h bioluminescence assay. Data represent the mean ± SEM of triplicates.

Statistical analysis was performed between *CD38*^{KO}*TRAC*^{KI} and *CD38*$^{\mathrm{KO/KI}}$EF1α groups. **(C)** Cytokine secretion by CAR-T cells from the two other donors was detected after stimulation by target cells. Data represent the mean ± SEM of triplicates. **(D)** FACS analysis of exhaustion makers (PD-1 and LAG-3) on CAR-T cells stimulated 1, 2, or 3 times by target cells. *P < 0.05, **P < 0.01, ***P < 0.001, and ****P < 0.0001; ns, not significant.

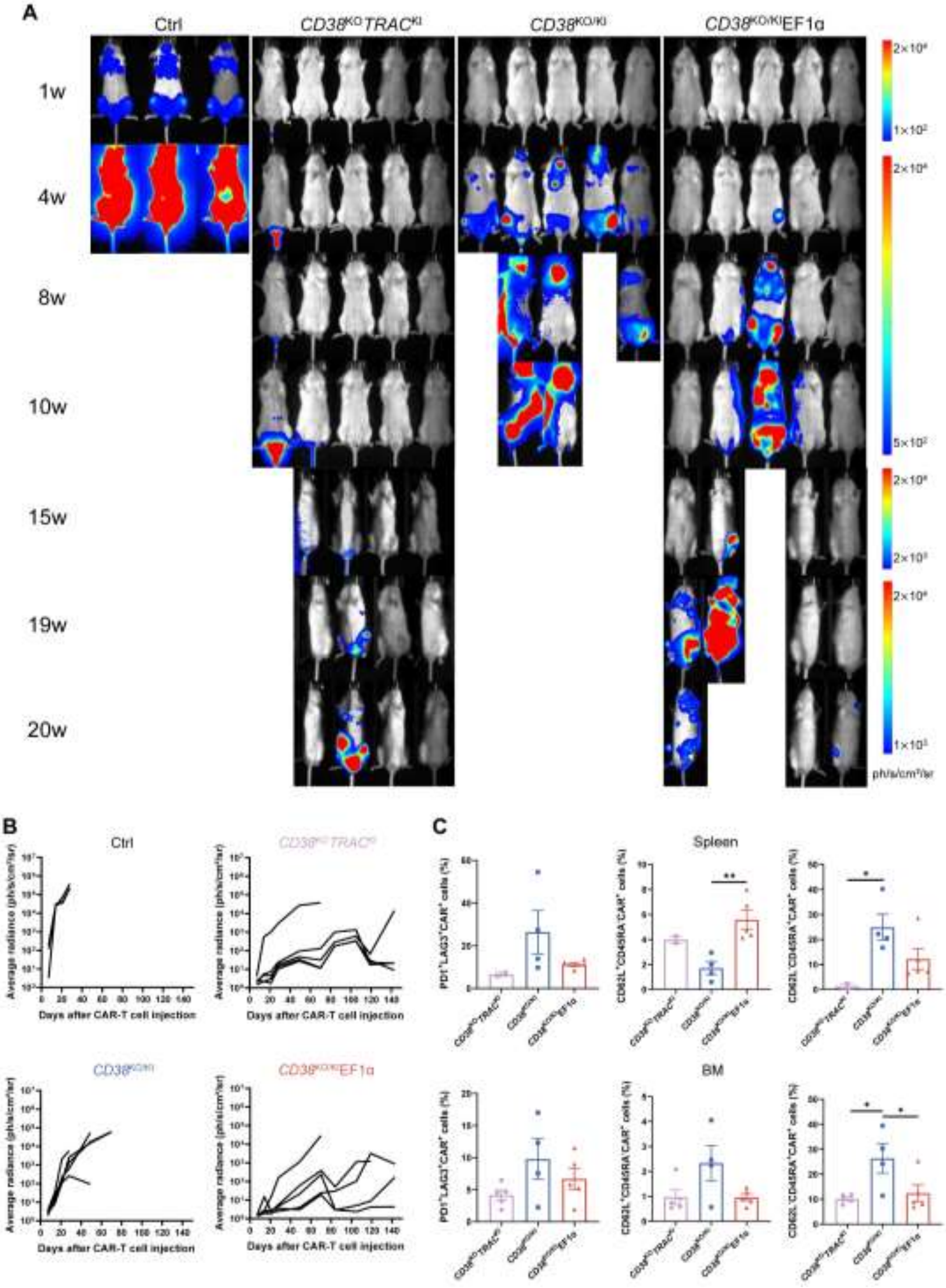

Figure S4: *CD38*$^{\mathrm{KO/KI}}$EF1α and *CD38*^{KO}*TRAC*^{KI} CAR-T cells showed better anti-tumor activity than *CD38*$^{\mathrm{KO/}}$

^{KI} **CAR-T cells *in vivo*. (A)** Representative bioluminescence imaging (BLI) results of tumor burden are shown. Control refers to treated with untransduced (UT) T cells (Donor 2). **(B)** The tumor burden (average radiance) of mice in each group was shown (Donor 2). **(C)** The phenotypes of CAR T cells in mice's bone marrow and spleen 49 d after CAR-T infusion, as demonstrated by the percentages of T_{CM} and T_{EFF} cells (Donor 1). Data were shown as mean ± SEM. *P < 0.05, **P < 0.01, ***P < 0.001, and ****P < 0.0001; ns, not significant.

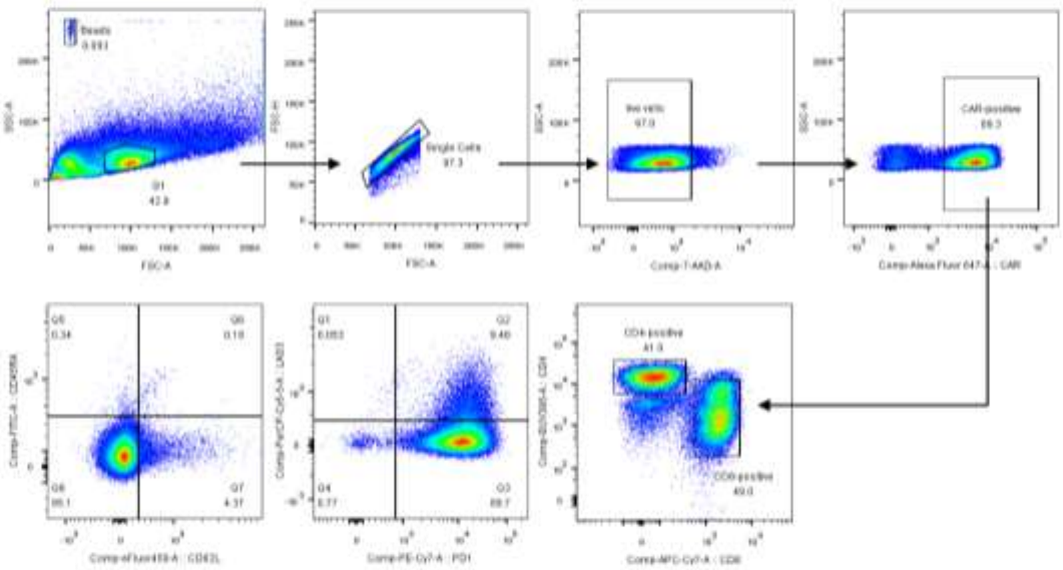

Figure S5: Gating strategy used to analyze CAR-T cells obtained from the bone marrow of treated mice. Representative flow cytometric analysis of *CD38*^{KO}*TRAC*^{KI} CAR-T cells on day 49 post CAR-T cell infusion (Donor 1).

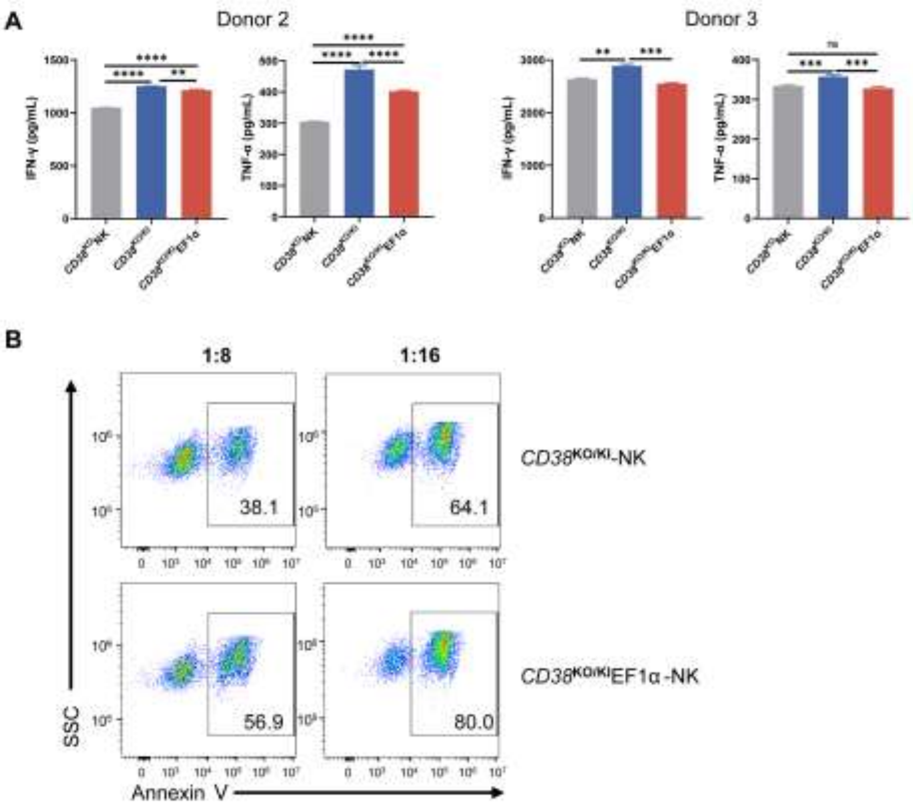

Figure S6: *In vitro* functional assays of CAR-NK cells. (A) Cytokine secretion by CAR-NK cells was detected after stimulation with Jurkat target cells (Donor 2 and Donor 3 in addition to Donor 1 in Fig. 6D). Data represent the mean ± SEM of triplicates. **(B)** CAR-NK cells upon stimulation for 24h were measured by surface Annexin V binding**.** *P < 0.05, **P < 0.01, ***P < 0.001, and ****P < 0.0001; ns, not significant.

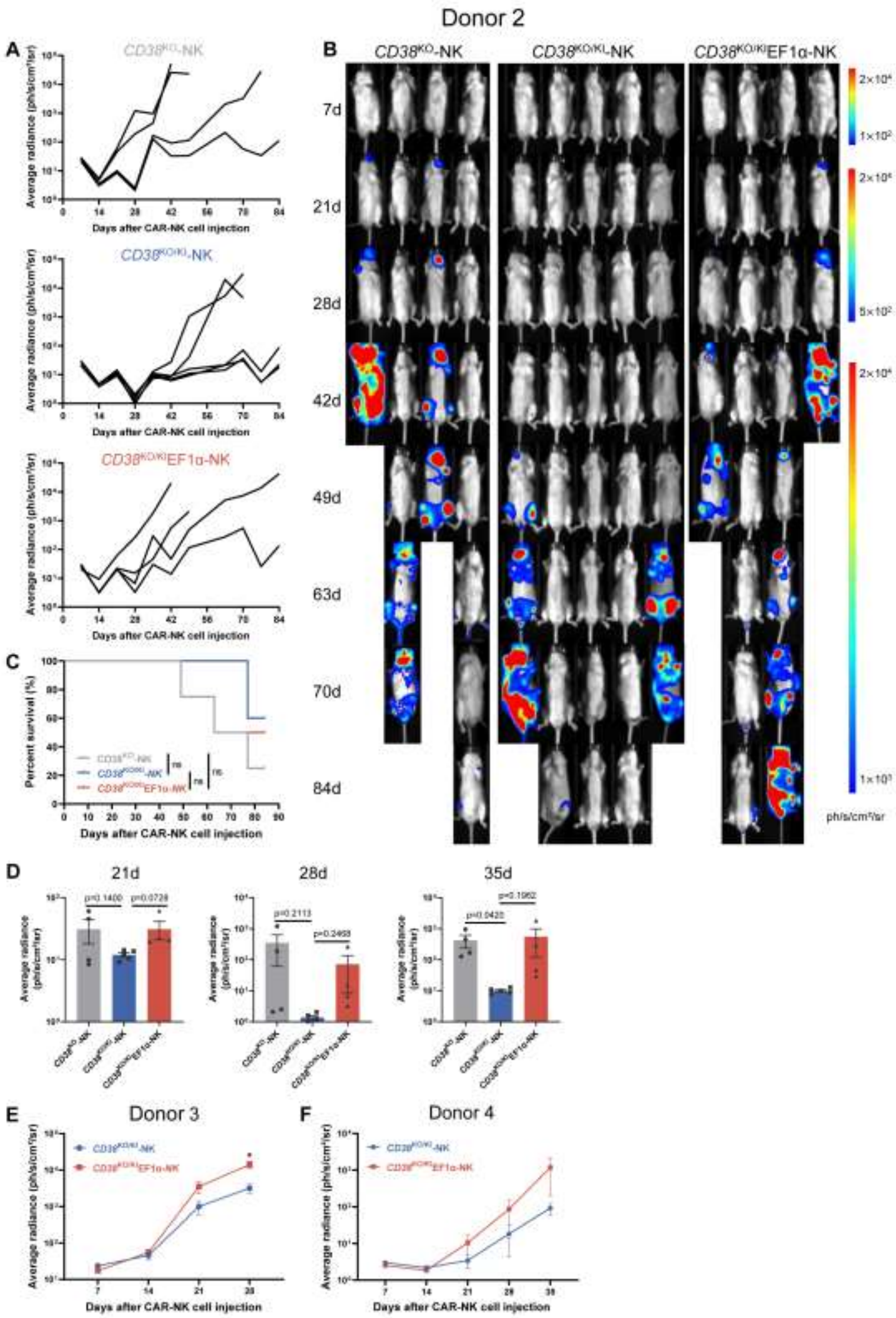

Figure S7: *In vivo* antitumor activity of CAR-NK cells. (A-D) 9×10^6 CD38 CAR-NK cells derived from Donor 2 were injected. **(A)** Tumor burden (average radiance) of mice in each group is shown. **(B)** Representative bioluminescence imaging (BLI) results of tumor burden are shown. **(C)** Kaplan-Meier survival curve of mice treated with *CD38*^{KO} NK cells or CD38 CAR-NK cells. **(D)** The tumor

burden (average radiance) of mice in each group at 21d, 28d and 35d after treatment was shown. Data were shown as mean ± SEM. **(E)** 3×10^6 CD38 CAR-NK cells derived from Donor 3 were injected and kinetics of tumor progression (average radiance) was evaluated by bioluminescence imaging. Data were shown as mean ± SEM. **(F)** 6.4×10^6 CD38 CAR-NK cells derived from Donor 4 were injected and kinetics of tumor progression (average radiance) was evaluated by bioluminescence imaging. Data were shown as mean ± SEM. *P < 0.05, **P < 0.01, ***P < 0.001, and ****P < 0.0001; ns, not significant.

Figure S8: Analysis of CAR-NK cells obtained from the peripheral blood, spleen and bone marrow of

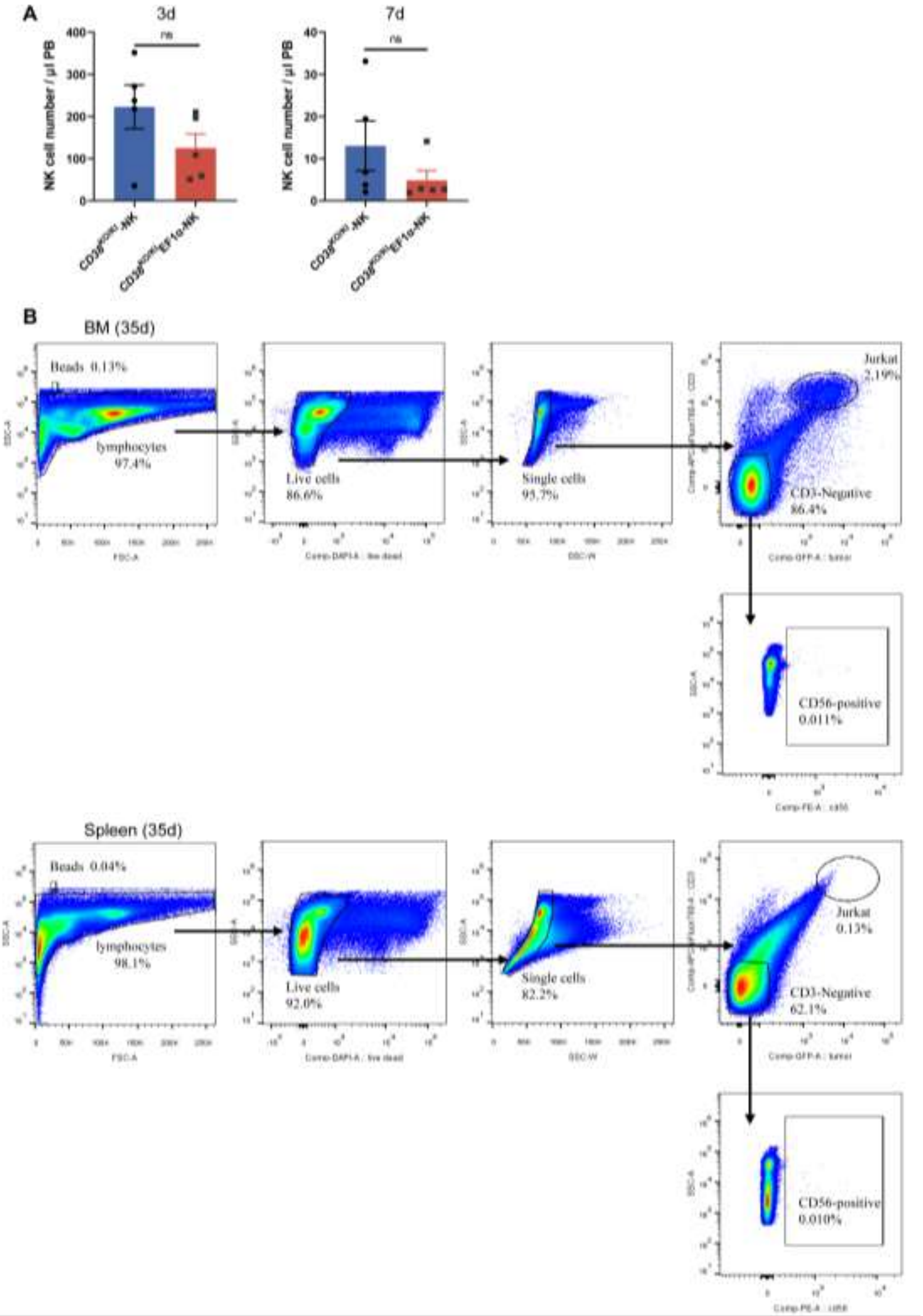

treated mice. (A) Peripheral blood (PB) of mice was collected on day3 and day7 post CAR-NK cells infusion and analyzed via flow cytometry. **(B)** Representative flow cytometric analysis of residual NK cells on day35 post CAR-NK cells infusion.

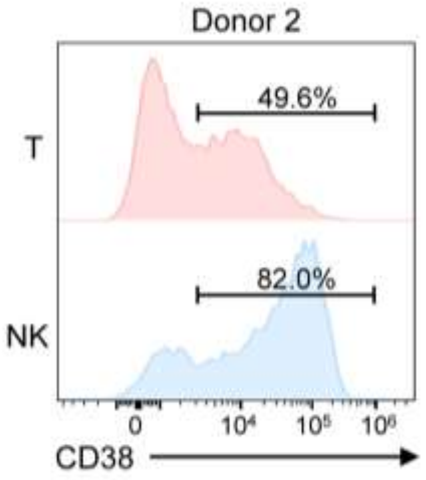

Figure S9: T and NK cells displayed distinct expression levels of CD38. Relative expression of CD38 on unactivated T and NK cells. CD38 expression of T and NK cells isolated from the peripheral blood without activation, as measured by flow cytometry and presented as a representative histogram (Donor 2 in addition to Donor 1 from Fig. 7A).

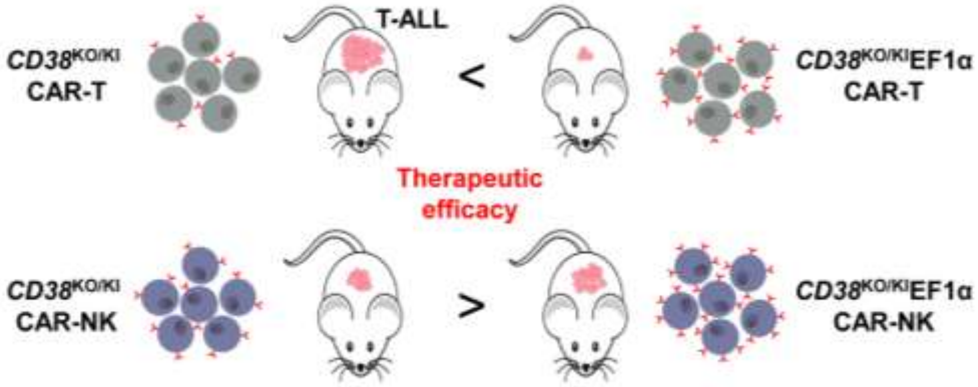

Figure S10: The schematic depicts the relationship between CD38 CAR expression levels of CAR-T/NK cells generated by our "2-in-1" strategy and their therapeutic efficacy.

Supplementary Tables

Table S1. Source and affinity of selected CD38 antibodies

Name	Source	KD (nM)
3079	Human	0.6
056	Mouse	3.4
Ab79	Human	5.5
